# Molecular epidemiological surveillance of monkeypox virus in Indonesia from 2023 to 2024

**DOI:** 10.1017/S0950268825100253

**Published:** 2025-06-30

**Authors:** Dhihram Tenrisau, Tri Bayu Purnama, Mulyanti Ayu Wulandari Maulana, Rizqy Fauzia Ahsani, Happy Kusuma Mulya, Yustinus Maladan, Listiana Azizah, Gerald Bagus A. Caloh, Firdaus Kasim

**Affiliations:** 1 Public Health Literature Club (PHLC), Yogyakarta, Indonesia; 2 Bioinformatics Research Center, Indonesia Bioinformatics and Biomolecular, Malang, Indonesia; 3 Faculty of Public Health, Universitas Islam Negeri Sumatera Utara Medan, Medan, Indonesia; 4 Division of International Health (Public Health), Graduate School of Medical and Dental Sciences, Niigata University, Niigata, Japan; 5 Ministry of Health, Infection Emerging Working Group, Directorate Surveillance and Quarantine, Indonesia; 6 Eijkman Research Center for Molecular Biology, National Research and Innovation Agency, Jl. Raya Bogor KM 46, Cibinong, Jawa Barat, Indonesia; 7 Nuffield Department of Medicine, University of Oxford, Oxford, UK

**Keywords:** molecular epidemiology, monkeypox, transmission, surveillance

## Abstract

Monkeypox (mpox) has re-emerged as global public health concern including in several non-endemic countries. This study aims to characterize monkeypox virus (MPXV) genomes in Indonesia, to explore viral evolution and transmission. Genomic analysis was conducted on 53 isolates from Indonesian mpox patients between 2023 and 2024. All sequences belonged to Clade IIb, with identified sub-clades including A.1.1, B.1, B.1.3, and C.1 – of which C.1 became dominant during this period. Out of 87 mpox-confirmed cases, 60.9% (53/87) were successfully sequenced and submitted to the Global Initiative on Sharing All Influenza Data (GISAID). The majority of cases in Indonesia occurred among males (95.4%), men who have sex with men (59.8%), and people living with HIV/AIDS (71.3%). Notably, a large portion of cases had no travel history, suggesting local transmission. Initially, only clade IIb (B.1) was detected in October 2022. By August 2023, lineage diversity had increased, with B.1.3 and C.1 emerging as the predominant sub-clades. A time–calibrated phylogenetic tree revealed genetic relatedness and shared ancestry within clade IIb. Integrating genomic and epidemiological data offers valuable insights to improve mpox surveillance and public health response in Indonesia and the broader region

## Introduction

Monkeypox (mpox), caused by monkeypox viruses (MPXV), is currently re-emerging as a viral zoonotic disease, driven by its ability to transmit from animals to humans, conversely. On 14 August 2024, the World Health Organization (WHO) declared the mpox outbreak a Public Health Emergency of International Concern (PHEIC), following reports of 18,737 cases and 541 deaths across 14 countries. However, in 2022, mpox cases began to appear in Europe, signalling the start of a broader geographical spread [1, 2]. By July 2022, the disease had spread to parts of Asia, with Southeast Asia experiencing fluctuating case numbers from May to September 2023 [3]. This resurfacing disease gained worldwide attention when an outbreak occurred in several non-endemic countries in May 2022 [4]. As of October 2024, the cumulative number of mpox cases in the Association of Southeast Asian Nations (ASEAN) varied, with Thailand reporting the highest total (858 cases), followed by Vietnam (203 cases), Indonesia (88 cases), Singapore (67 cases), the Philippines (32 cases), and Malaysia (10 cases). Additionally, deaths were reported in Thailand (10 deaths) and Vietnam (8 deaths) [5].

In Indonesia, the first case of mpox was reported in October 2022, identified as an imported case from travelling to Western European countries. Subsequent cases were identified in several regions, including reports from DKI Jakarta, Banten, West Java, East Java, Riau Islands, and Yogyakarta [3, 6, 7]. As of September 2024, Indonesia had reported a total of 88 mpox-positive/confirmed cases [3]. The clade IIb MPXV outbreak appears to have been predominantly driven by sustained human-to-human transmission, primarily through intimate contact within the men who have sex with men (MSM) community [8].

Global mpox surveillance initiatives, along with their genomic approaches, are essential for advancing knowledge of the disease, mapping the evolutionary path of the virus, and exploring its genetic variability. Recent studies have highlighted the importance of integrating genomic surveillance with epidemiological data to effectively track mpox transmission and identify potential outbreak sources [9, 10]. These efforts offer valuable insights into the virus’s genetic makeup and potential phenotypic associations. The collected data have played a key role in tracing the evolution of MPXV, investigating the emergence of new viral lineages, and tracking both local and global transmission patterns. This study intends to describe the epidemiology and genomic surveillance of mpox cases in Indonesia. In addition, this study explored the genetic relationships among MPXV isolates from Indonesia and other Southeast Asian countries through phylogenetic analysis using whole genome sequencing. The objective was to uncover valuable insights into the virus’s transmission dynamics and patterns, highlighting the distinctive characteristics of isolates from Southeast Asian countries.

## Methods

### Study design

We conducted a cross-sectional, descriptive study involving genomic sequence analysis of real-time of confirmed cases in Indonesia between January 2023 and October 2024. These samples were collected as part of a national surveillance program established by the Ministry of Health, Indonesia, under which informed consent was waived [11].

### Epidemiology data

The epidemiology investigation of mpox cases was collected by the Infection Emerging Working Group, Ministry of Health, Indonesia. A suspected case is characterized by the onset of a rash on the skin or mucous membranes, potentially accompanied by symptoms such as fever, chills, muscle aches, headaches, swollen lymph nodes, lower back pain, fatigue, proctitis, or a history of contact with a confirmed case within the preceding 21 days, along with recent travel to endemic regions [11]. The rash phase spans from the appearance of lesions to their shedding, typically lasting 14–28 days. In mpox cases, the rash usually begins 1–5 days after the onset of initial symptoms, initially presenting as fluid-filled vesicles that evolve into pustules before finally forming scabs [12]. Mpox cases are classified into two categories based on laboratory confirmation through polymerase chain reaction (PCR) testing: suspected or probable cases with negative PCR results are classified as discarded, while confirmed cases are those with a positive PCR test for the MPXV [11].

The mpox case definitions were categorized into two groups based on WHO guidelines [13]. The risk or prognosis became the basis of classification into mild and severe. A severe case is defined as meeting any of the following criteria. High-risk patients include children under 8 years old, pregnant women, people living with HIV (PLWH) not on antiretroviral treatment (ART) or who are immunocompromised, and patients with atopic dermatitis at risk of secondary infection. Severe cases may also involve complications, such as nausea, cervical lymphadenopathy (swollen lymph nodes in the neck), eye problems, hepatomegaly (enlarged liver), sepsis, dehydration, pneumonia, or loss of consciousness. Additionally, abnormal laboratory results indicating a severe case include having three or more of the following: high white blood cell count, elevated liver enzymes (Alanine aminotransferase (ALT), also known as SGPT and/or Aspartate aminotransferase (AST), also known as SGOT), or low blood urea nitrogen (BUN). Lastly, patients with more than 100 skin lesions are also categorized as severe cases [11]. Such laboratory findings align with prior studies highlighting the prognostic value of specific biomarkers in severe mpox cases [14].

The length of treatment is defined as the total number of days a patient spends from the clinical diagnosis of mpox to final status or discharge (recovered or death). In addition, the relationship between sub-clades and lineages was checked with Fisher’s exact test as the ‘distribution free’ method for the small sample size study [15].

### Sequences collection and processing

All sequences used in this study were obtained from the Global Initiative on Sharing All Influenza Data (GISAID), a platform recognized for supporting WHO Member States and public health authorities in expanding data sharing for pathogens with pandemic potential, including MPXV [16, 17]. Indonesia has contributed data to GISAID since October 2006, and its genomic data has been featured in various publications [18–20]. The data were accessed on 29 November 2024.

Metadata extracted from GISAID included location, date, and other tags; however, only the unique accession ID was used in this study and merged with epidemiological investigation data. Only Monkeypox virus sequences exceeding 190,000 bp and meeting quality standards were included, resulting in 54 eligible sequences. These sequences, representing mpox cases reported from primary healthcare facilities and hospitals across Indonesia, were uploaded to GISAID between 2022 and 2024. The details of sequences that were utilized can be seen in the table in Supplementary Material 10. Genome classification was performed using Nextclade. (https://github.com/nextstrain/mpox/blob/master/phylogenetic/defaults/clades.tsv).

Genome sequences were aligned using the multiple alignment software MAFFT version 7.490 (https://mafft.cbrc.jp/alignment/software/). After alignment inspection and correction in Aliview, the sequences were finalized for analysis, the ambiguous sequences from multiple alignment sequences will be compared in the following process. The maximum likelihood trees were inferred and reconstructed with IQ-TREE (http://www.iqtree.org/). Reconstruction was done under the best-fitting model of nucleotide substitution, as determined by ModelFinder in IQ-TREE. Ultrafast Bootstrap was used to estimate branch supports, with 1,000 replicates.

### Phylogenetic and molecular clock analysis

The Indonesian phylogenetic tree was generated from all of the sequence cases in Indonesia. Meanwhile, the ASEAN phylogenetic tree included the complete genome from mpox cases of other ASEAN countries in the GISAID repository. The ASEAN tree was rooted against the reference (Accession number: NC_063383.1). The ambiguous sequences were imputed by the consensus sequences. The molecular clock analysis began with a diagnostic test using TempEst (http://tree.bio.ed.ac.uk/software/tempest/), a genomic analysis tool that evaluates the clock-like behaviour of a dataset. TempEst performed root-to-tip linear regressions based on the phylogenetic tree.

The phylogeny calibrated-tree was generated with TreeTime (https://treetime.readthedocs.io/en/latest/) as the standard molecular clock. This package utilizes the Maximum Likelihood approach to infer evolution models and estimate molecular clock phylogenies and population size histories. The sensitivity analysis was performed by comparing molecular clock analysis with the trees with non-imputed sequences. A detailed description of the pipeline analysis is available in Supplementary Material 1 accompanying this study. Ethical approval was obtained from the Ethical Clearance Department of Universitas Islam Sumatera Utara, Medical Faculty, with the reference number: 100/EC/KEP/UISU/XII/2024.

## Result

A total of 87 mpox PCR-positive results were included in this study, with 61% (53 of 87) of these cases reported with whole genome sequencing (WGS) data. The cases in Indonesia were dominated by the males (95.4%), males who have sex with males (59.8%), and people living with HIV/AIDS (71.3%). Greater Jakarta, as the biggest city network and capital of Indonesia, was potentially epicentrum of the mpox cases in Indonesia (88.5%). Mpox cases were predominantly reported at public health centres, accounting for 67.8% (59 cases) of the total. Notably, 26 cases (29.89%) classified as severe did not require hospital treatment, as their clinical condition permitted self-isolation.

**Figure 1.**
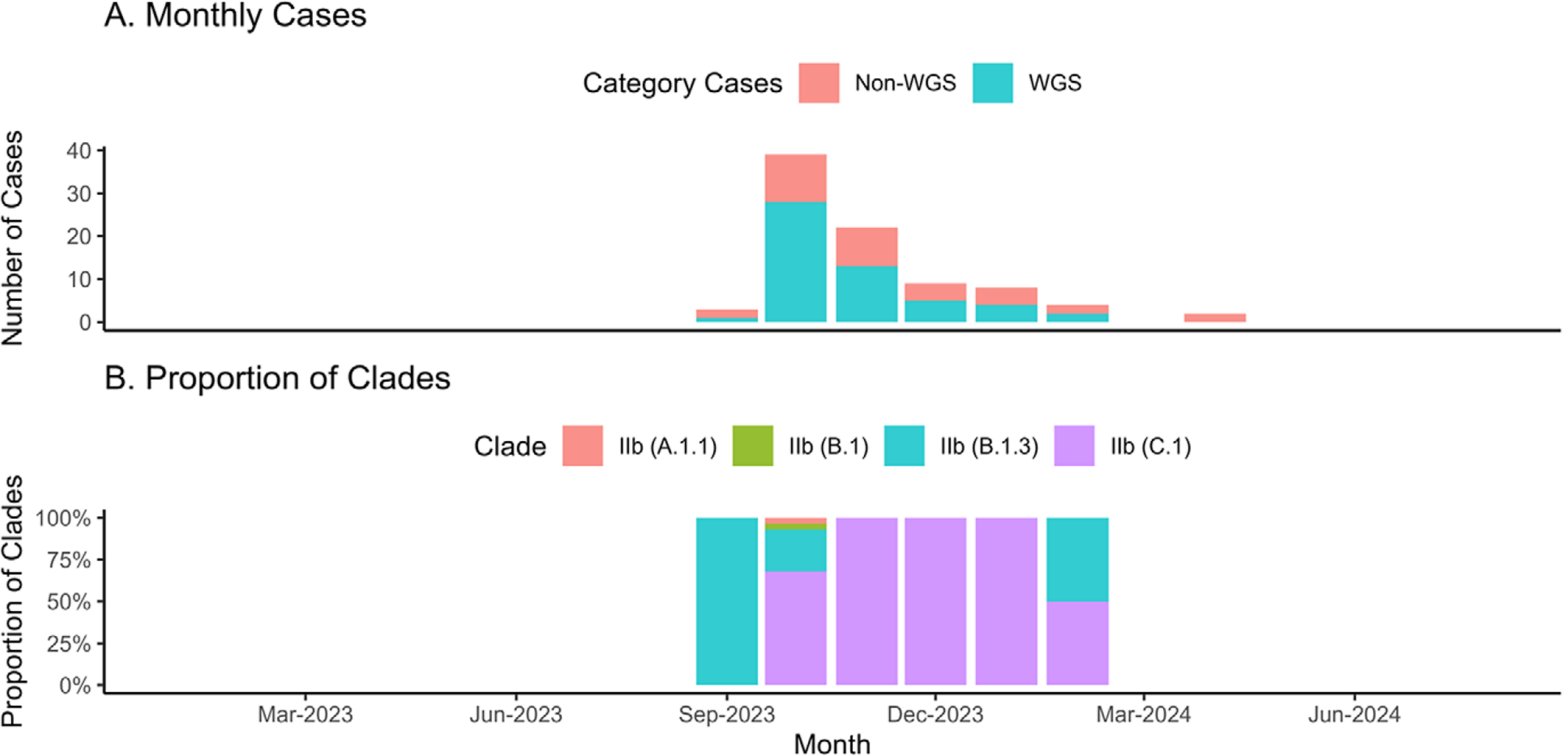
The monthly epidemic curve of mpox cases during study period in Indonesia. The epi-curve of mpox cases monthly in Indonesia among whole genome sequencing and non- whole genome sequencing samples (1A). Meanwhile, Figure 1B illustrates the trend of sub-clade among the whole genome sequencing samples.

The mpox cases in Indonesia were also dominated by mild cases (51.7%), with the average duration of treatment being 30 days (SD = 14.3–45.9 days). All patients recovered, including the three cases without symptoms (3.4%). A total of 79 cases (90.8%) were reported without travel history (Table 1). The cases were divided into the group that proceeded to Whole Genome Sequencing (WGS) and without WGS (non-WGS). Figure 1a illustrates the monthly epidemic curve of mpox cases, distinguishing between those analysed through WGS and non-WGS. There were two outbreak periods: 2022 and 2023, as well as the pandemic status revoked by Indonesia’s government, and the surge of tourist visits (Supplementary Material 2). A notable surge in cases was observed in August 2023, followed by a gradual decline, with both WGS and non-WGS cases contributing to the peak. Of the 87 mpox cases reported, 53 underwent WGS, while the remaining 34 were not sequenced.

**Table 1.** Distribution of epidemiology and clinical data of all mpox cases between 2023 and 2024

	Total (*N* = 87)	WGS (*N* = 53)	Non-WGS (*N* = 34)
Sex			
Male	83 (95.4%)	50 (94.3%)	33 (97.1%)
Female	4 (4.6%)	3 (5.7%)	1 (2.9%)
Age group			
20–64	86 (98.9%)	53 (100%)	33 (97.1%)
13–19	1 (1.1%)	0 (0%)	1 (2.9%)
Sexual orientation			
MSM	52 (59.8%)	34 (64.2%)	18 (52.9%)
Bisexual	22 (25.3%)	11 (20.8%)	11 (32.4%)
Heterosexual	13 (14.9%)	8 (15.1%)	5 (14.7%)
Travel history			
No	79 (90.8%)	47 (88.7%)	32 (94.1%)
Yes, Domestic	3 (3.4%)	2 (3.8%)	1 (2.9%)
Yes, East Asia	2 (2.3%)	1 (1.9%)	1 (2.9%)
Yes, Southeast Asia	2 (2.3%)	2 (3.8%)	0 (0%)
Yes, Mediterranean	1 (1.1%)	1 (1.9%)	0 (0%)
Origin report			
PHC	59 (67.8%)	38 (71.7%)	21 (61.8%)
Hospital	28 (32.2%)	15 (28.3%)	13 (38.2%)
Location			
The Greater Jakarta	77 (88.5%)	50 (94.3%)	27 (79.4%)
Outside Greater Jakarta	10 (11.5%)	3 (5.7%)	7 (20.6%)
Severity criteria			
Mild	45 (51.7%)	29 (54.7%)	16 (47.1%)
Severe	39 (44.8%)	23 (43.4%)	16 (47.1%)
Asymptomatic	3 (3.4%)	1 (1.9%)	2 (5.9%)
Clade			
IIb (C.1)	42 (48.3%)	42 (79.2%)	0 (0%)
IIb (B.1.3)	9 (10.3%)	9 (17.0%)	0 (0%)
IIb (A.1.1)	1 (1.1%)	1 (1.9%)	0 (0%)
IIb (B.1)	1 (1.1%)	1 (1.9%)	0 (0%)
Non-detected	34 (39.1%)	0 (0%)	34 (100%)
HIV status			
Yes	62 (71.3%)	41 (77.4%)	21 (61.8%)
No	25 (28.7%)	12 (22.6%)	13 (38.2%)
Isolation status			
Self isolation	66 (75.9%)	40 (75.5%)	26 (76.5%)
Hospital isolation	21 (24.1%)	13 (24.5%)	8 (23.5%)
Length of treatment (days)			
Mean (SD)	30.1 (15.8)	31.0 (15.4)	28.7 (16.5)
Median [Min, Max]	27.0 [10.0, 109]	27.0 [10.0, 109]	24.0 [14.0, 103]
Final status			
Recovered	87 (100%)	53 (100%)	34 (100%)

Abbreviations: WGS, whole genome sequencing; ART, antiretroviral treatment; MSM, men who have sex with men; HIV, human immunodeficiency virus; SD, standard deviation.

Sequencing achieved complete genome coverage, with an average read depth of 1,315.302× and a mean sequence length of 197,193 bases for the virus strains (Supplementary Material 4). All sequenced genomes from Indonesia were identified as belonging to Clade IIb. Figure 1b highlights the evolving clade trend: in October 2022, only clade IIb (B.1) was detected. By August 2023, clade diversity expanded, with clades IIb (B.1.3) and IIc (C.1) emerging as the predominant types in subsequent months. Half of the cases were classified as mild, with the distribution showing slight variation across lineages. Severe cases were more prevalent in B.1.3, while asymptomatic cases were rare across all sub-clades. Nearly all individuals received treatment for 14 days or more, regardless of lineage. Most individuals were HIV-positive across all sub-clades, with B.1 consisting solely of non-ART cases and A.1.1 showing complete ART coverage. Despite these patterns, the lineages or sub-clades showed no statistically significant associations (p-value >0.05) with clinical criteria such as severity, length of treatment, HIV status, sexual orientation, or ART treatment (Table 2).

**Table 2.** Descriptive summary of the relationships between different Clades (IIb sub-clade or lineages) and clinical conditions, along with *p*-values from non-parametric Fisher’s exact test

	Total (*N* = 53)	IIb (A.1.1) (ref) (*N* = 1)	IIb (C.1) (*N* = 42)	IIb (B.1.3) (*N* = 9)	IIb (B.1) (*N* = 1)	P-value
Severity criteria						
Mild	29 (54.7%)	25 (59.5%)	3 (33.3%)	1 (100%)	0 (0%)	0.3065
Severe	23 (43.4%)	16 (38.1%)	6 (66.7%)	0 (0%)	1 (100%)	
Asymptomatic	1 (1.9%)	1 (2.4%)	0 (0%)	0 (0%)	0 (0%)	
Length of treatment						
≤ 14 days	1 (1.9%)	1 (2.4%)	0 (0%)	0 (0%)	0 (0%)	1
≥ 14 days	52 (98.1%)	41 (97.6%)	9 (100%)	1 (100%)	1 (100%)	
Sexual.orientation						
Heterosexual	8 (15.1%)	7 (16.7%)	1 (11.1%)	0 (0%)	0 (0%)	1
MSM	34 (64.2%)	26 (61.9%)	6 (66.7%)	1 (100%)	1 (100%)	
Bisexual	11 (20.8%)	9 (21.4%)	2 (22.2%)	0 (0%)	0 (0%)	
HIV						1
No	12 (22.6%)	10 (23.8%)	2 (22.2%)	0 (0%)	0 (0%)	
Yes	41 (77.4%)	32 (76.2%)	7 (77.8%)	1 (100%)	1 (100%)	
ART_treatment						
Non-ART	7 (13.2%)	4 (9.5%)	2 (22.2%)	0 (0%)	1 (100%)	0.4542
ART	33 (62.3%)	27 (64.3%)	5 (55.6%)	1 (100%)	0 (0%)	
Treatment Interruption	1 (1.9%)	1 (2.4%)	0 (0%)	0 (0%)	0 (0%)	
Non-HIV	12 (22.6%)	10 (23.8%)	2 (22.2%)	0 (0%)	0 (0%)	

Abbreviations: ART: Antiretroviral treatment, MSM: Men who have sex with men, HIV: Human Immunodeficiency Virus.

**Figure 2.**
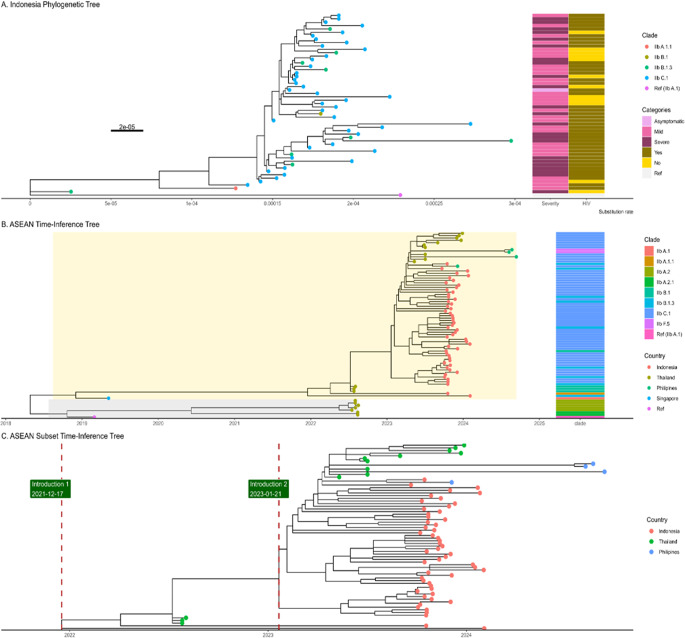
The A, displays a phylogenetic tree of cases from Indonesia, with the x-axis representing the substitution rate with epidemiological data. In contrast, the B. figure illustrates a time–calibrated phylogenetic tree of sequences from ASEAN countries, indicating that the most recent common ancestor (TMRCA) dates to approximately July 2018. The B tree diverged into two groups or branches: Group 1 (yellow shade) and Group 2 (gray shade). Also, The B tree tips are also divided into clade subgroups or lineages. The C. is the ASEAN Subset where the green label and red line were positioned at the time of introduction estimation (December 2021 and January 2023). Both ASEAN and Indonesia’s tree were rooted against reference (NC_063383.1).

The phylogenetic trees of Indonesia show a very weak and negative molecular clock signal (R-squared = 0.039 and 0.31), meanwhile, the ASEAN tree depicts a moderate molecular clock signal (R-squared = 0.4) (Supplementary Materials 5 and 6). The ASEAN tree imputation of ambiguous sequences generated a slightly lower molecular clock signal (R-square = 0.39). This sensitivity analysis suggested that the imputation of the ambiguous sequences might not alleviate the temporal relationship for a time-calibrated phylogenetic tree. The phylogenetic tree highlights MPXV genome sequences from several ASEAN countries, including Indonesia. Indonesian sequences, marked in yellow, dominate the tree and are dispersed across multiple branches, reflecting genetic diversity within the region. Most Indonesian sequences cluster closely, indicating a shared ancestry and potential localized transmission (Figure 2).

The ASEAN tree, without imputation as the best tree for molecular clock, showed several sequences that became outliers that reduce the root-to-tip regression. In order to get a more proper molecular signal, several outliers were removed from the tree (Supplementary Material 7). As a result, the molecular signal became stronger after removing seven outlier tips (Supplementary Material 8). The time-scaled phylogenetic tree depicts the Time of Most Recent Common Ancestor (TMRCA) in July 2018, when the tree diverged into two branches (Figure 2b). All of Indonesia’s cases were in Group 1 (yellow shade). Based on the tree, it is possible that the mpox was introduced in Indonesia in December 2021. In addition, there were possibly multiple introductions of mpox, as shown in Figure 2c, which depicts two clusters of tips from Indonesia.

## Discussion

This study identified the TMRCA, suggesting the likely time of mpox introduction in Indonesia, and reinforced previous findings regarding the populations at highest risk for mpox transmission. Those groups indicate sexual activity as a risk factor of mpox transmission, as mentioned in previous studies. Moreover, the stigma, discrimination, and social exclusion among HIV-MSM in Indonesia’s urban area potentially resulted in health inequalities and high-risk behaviours of mpox transmission [21, 22].

The mpox cases in Indonesia were mostly detected in the Public Health Centre (69%), as the first-level health facility for non-serious health conditions. This indicates that the mpox did not show serious clinical conditions. However, the HIV patients without ART potentially develop severe conditions, where 84% (11 patients from 13) of them are classified as severe. This suggests that the antiviral treatment should be considered as a component of case management, particularly for patients with severe HIV [23]. In addition, the mean length of treatment of all cases was 30 days (SD = 14.3–45.7 days), which doubled from the previous finding. This might be caused by prolonged PCR positivity, as mentioned in the previous study [24]. Besides, the high proportion of self-isolation might have contributed to the prolonged treatment.

Meanwhile, the first case of mpox in Indonesia is the B.1 clade that was reported in the first case with a Western European travel history. In fact, this lineage became the majority lineage across Europe and the Americas and has been an endemic since 2022 [25]. It is aligned with the findings where this clade was spread by the travel activity and then shifted to the local transmission in the risk group [26]. Consistent with the previous studies, the high proportion of non-travel population indicates the underreported cases, and the cases have been rapidly spreading in the community after the first case.

The international travellers were an important factor in the worldwide spread of mpox. Indonesia, as part of Southeast Asia, is a popular tourist destination, where the potential for silent spread appears. This condition might be explained by the two periods of outbreaks, which occurred during the high season of tourist visits in Indonesia (see Supplementary Materials). Consistent with the previous studies, the mpox cases in Indonesia were predominantly reported from urban areas, especially in the Greater Jakarta, the biggest urban area in Indonesia [27, 28].

The phylogenetic tree of Indonesian MPXV sequences, rooted with the earliest cases, shows that clade IIb B.1 serves as the ancestral lineage, with clades B.1.3 and C.1 emerging later and dominating recent branches. These sub-clades B.1.3 and C.1 appear to share a recent common ancestor because they emerge from the same branch in the phylogenetic tree, particularly among ASEAN countries. The dominance of C.1 among other sub-clades indicates that this sub-clade might be circulating domestically in Indonesia. Previous study also revealed that the lineages of clade IIb, gene content A and B.1 were similar. The mutation rate in Indonesia was slower (0.00001) than the previous study, which reported the substitution rate span from 0.02 to 0.038 [28–30]. From the time inference of the phylogenetic tree, it was found that the TMRCA was estimated in December 2021 (2021-2112-17). A previous study also showed that the 2022 outbreak of mpox was estimated to have begun in July 2021 [31]. The phylogenetic time tree also might suggest the multiple introductions (for sub-clade C.1 and B.1), as well as the study in Ireland [32]. This study also found that less diversification among lineages occurred where the unclustering the lineages in the phylogenetic tree. The similarities also possibly contributed to weak molecular clock signals. Previously, the study of clade IIb B.1 also reported a lack of a positive association between sequence divergence and sampling dates due to very low genetic variability [25].

A limitation of this study was representing the cases with WGS for only 61% of all mpox cases in Indonesia. Both of those factors possibly contributed to the accuracy of the phylogenetic and time-calibrated trees. This can be traced to the underestimate of TMRCA timescale phylogenetic tree: 2007.4 (2007-2105-27) and the slower substitution rate [33]. Besides, there was also the potential of underreporting cases that might have caused the high proportion of mild symptoms among cases [34]. A previous study in the Netherlands illustrates that the mpox patients tend to do self-isolation and self-diagnosis [35]. The patients tend to choose those medications due to a lack of knowledge, stigma, and discrimination. Studies in the United States and China enhance the community-based surveillance to detect, prevent, and control at the community level [36, 37]. Despite the limitation, accelerated contact tracing and genomic analysis remain a critical tool for informing public health responses to the mpox epidemic. Genomic surveillance supports our understanding of the virus and complements traditional contact tracing efforts or epidemiology data [10]. Combining those two methods could help with effective systematic quarantine to prevent and trace the transmission in the community, and support the vaccination program for high-risk populations. Strengthened public health measures, tailored to Indonesia’s unique context, are essential to mitigating the impact of mpox and preventing future outbreaks [38, 39].

## Supporting information

10.1017/S0950268825100253.sm001 Tenrisau et al. supplementary material Tenrisau et al. supplementary material

## Data Availability

All the data of sequences have been obtained in GISAID, the details are available in Supplementary Material 10 and the code can be accessed on the GitHub repository at https://github.com/Dhihram/Indo-MPX.

## Supplementary material

The supplementary material for this article can be found at http://doi.org/10.1017/S0950268825100253.
