# Supplementary material for: Molecular epidemiological surveillance of monkeypox virus in Indonesia from 2023 to 2024
Source: Epidemiol Infect. 2025 Jun 30;153:e79. doi: 10.1017/S0950268825100253 (PMC12247005; doi:10.1017/S0950268825100253)
Supplement: Tenrisau et al. supplementary material [file S0950268825100253sup001.docx]

**Supplementary material 1.** The flowchart of the data process and analysis. This analysis adopting Nextstrain pipeline for mpox


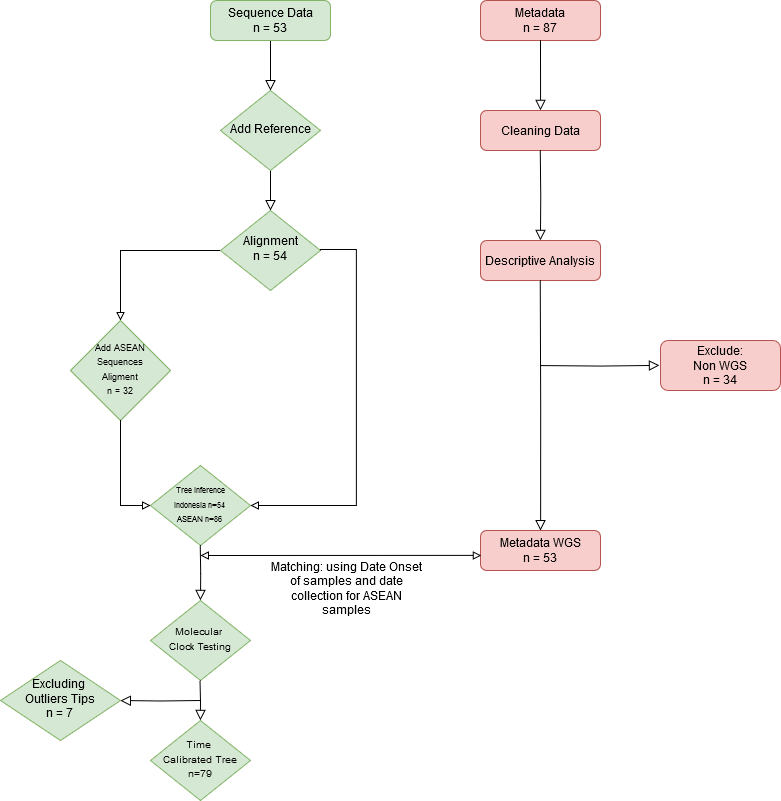


**Supplementary material 2.** The international tourist visiting trend in Indonesia from 2022 to 2024. The annual high season was reported between August and December where the periods of mpox outbreaks and first cases occurred. The outbreak was occurred 4 months after COVID-19 pandemic status was revoked (21-06-2023). The time introduction that estimated from molecular clock reported after tourist high seasons of 2021 and 2023. The data were scrapped from Indonesia’s Central Bureau of Statistics.

*
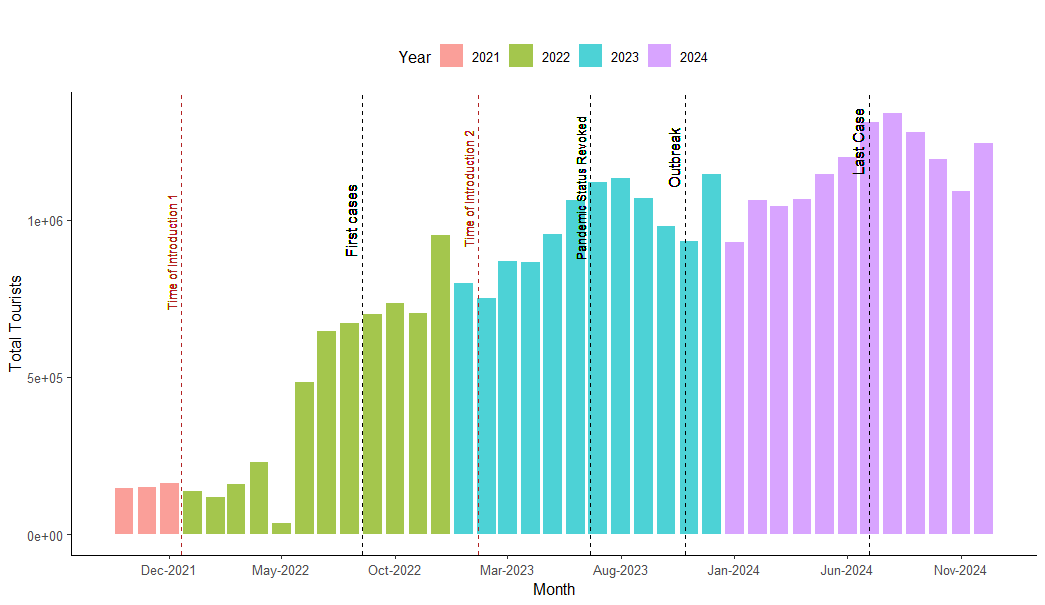
*

**Supplementary material 3.** The best model of every tree in analysis. The model selection was performed by ModelFinder in IQ-TREE

| **Tree** | **Model** | **AIC** | **AICc** | **BIC** |
| --- | --- | --- | --- | --- |
| Best Model ASEAN Without Imputation | HKY+F+R5 | 532939.982 | 532940.323 | 534805.121 |
| Best Model ASEAN With Imputation | HKY+F+R5 | 528616.492 | 528616.636 | 529829.342 |
| Best Model Indonesia Without Imputation | HKY+F+I | 528769.032 | 528769.161 | 529910.538 |
| Best Model Indonesia With Imputation | HKY+F+R5 | 528616.492 | 528616.636 | 529829.342 |

**Supplementary material 4.** Comprehensive overview of mpox genome sequencing data: sequence identifiers, genome lengths, and coverage depth

| **Sequence** | **Accession ID** | **Length** | **Coverage** |
| --- | --- | --- | --- |
| hMpxV/Indonesia/JK-NIHRD-MP005/2023 | EPI_ISL_18436040 | 197,209 | 42x |
| hMpxV/Indonesia/JK-NIHRD-MP010/2023 | EPI_ISL_18436041 | 197,204 | 84x |
| hMpxV/Indonesia/JK-NIHRD-MP008/2023 | EPI_ISL_18463158 | 197,204 | 464x |
| hMpxV/Indonesia/JK-NIHRD-MP015/2023 | EPI_ISL_18463160 | 197,201 | 78x |
| hMpxV/Indonesia/JK-NIHRD-MP012/2023 | EPI_ISL_18463159 | 197,200 | 51x |
| hMpxV/Indonesia/JK-NIHRD-MP017/2023 | EPI_ISL_18463161 | 197,200 | 342x |
| hMpxV/Indonesia/JK-NIHRD-MP023/2023 | EPI_ISL_18467795 | 197,199 | 117 |
| hMpxV/Indonesia/JK-NIHRD-MP022/2023 | EPI_ISL_18467799 | 197,199 | 998x |
| hMpxV/Indonesia/JK-NIHRD-MP025/2023 | EPI_ISL_18467796 | 197,199 | 272x |
| hMpxV/Indonesia/JK-NIHRD-MP044/2023 | EPI_ISL_18467805 | 197,199 | 34x |
| hMpxV/Indonesia/JK-NIHRD-MP036/2023 | EPI_ISL_18467800 | 197,199 | 123x |
| hMpxV/Indonesia/JK-NIHRD-MP039/2023 | EPI_ISL_18467801 | 197,199 | 1226x |
| hMpxV/Indonesia/JK-NIHRD-MP035/2023 | EPI_ISL_18467804 | 197,199 | 19x |
| hMpxV/Indonesia/JB-NIHRD-MP041/2023 | EPI_ISL_18467808 | 197,199 | 105x |
| hMpxV/Indonesia/JK-NIHRD-MP081/2023 | EPI_ISL_18467798 | 197,199 | 115x |
| hMpxV/Indonesia/JK-NIHRD-MP061/2023 | EPI_ISL_18467802 | 197,199 | 17x |
| hMpxV/Indonesia/BT-NIHRD-MP059/2023 | EPI_ISL_18467794 | 197,199 | 300x |
| hMpxV/Indonesia/JK-NIHRD-MP055/2023 | EPI_ISL_18467797 | 197,199 | 46x |
| hMpxV/Indonesia/JK-NIHRD-MP080/2023 | EPI_ISL_18467806 | 197,199 | 142x |
| hMpxV/Indonesia/JK-NIHRD-MP085/2023 | EPI_ISL_18467803 | 197,199 | 3462x |
| hMpxV/Indonesia/JK-NIHRD-MP088/2023 | EPI_ISL_18467807 | 197,199 | 35x |
| hMpxV/Indonesia/JK-NIHRD-MP109/2023 | EPI_ISL_18642356 | 197,199 | 340x |
| hMpxV/Indonesia/JK-NIHRD-MP114/2023 | EPI_ISL_18642357 | 197,199 | 68x |
| hMpxV/Indonesia/JK-NIHRD-MP118/2023 | EPI_ISL_18642358 | 197,199 | 85x |
| hMpxV/Indonesia/JK-NIHRD-MP147/2023 | EPI_ISL_18642359 | 197,199 | 66x |
| hMpxV/Indonesia/JK-NIHRD-MP156/2023 | EPI_ISL_18642360 | 197,199 | 140x |
| hMpxV/Indonesia/JK-NIHRD-MP177/2023 | EPI_ISL_18642361 | 197,199 | 190x |
| hMpxV/Indonesia/JB-NIHRD-MP205/2023 | EPI_ISL_18642362 | 197,199 | 187x |
| hMpxV/Indonesia/JK-NIHRD-MP209/2023 | EPI_ISL_18642363 | 197,199 | 205x |
| hMpxV/Indonesia/JK-NIHRD-MP218/2023 | EPI_ISL_18642364 | 197,199 | 108x |
| hMpxV/Indonesia/BT-NIHRD-MP231/2023 | EPI_ISL_18642365 | 197,199 | 89x |
| hMpxV/Indonesia/JK-NIHRD-MP159/2023 | EPI_ISL_18798834 | 197,199 | 137x |
| hMpxV/Indonesia/JK-NIHRD-MP225/2023 | EPI_ISL_18798835 | 197,199 | 78x |
| hMpxV/Indonesia/JK-NIHRD-MP235/2023 | EPI_ISL_18798836 | 197,199 | 172x |
| hMpxV/Indonesia/JK-NIHRD-MP246/2023 | EPI_ISL_18798837 | 197,199 | 489x |
| hMpxV/Indonesia/JB-NIHRD-MP254/2023 | EPI_ISL_18798839 | 197,197 | 585x |
| hMpxV/Indonesia/JB-NIHRD-MP255/2023 | EPI_ISL_18798840 | 197,196 | 45x |
| hMpxV/Indonesia/JK-NIHRD-MP252/2023 | EPI_ISL_18798838 | 197,196 | 31x |
| hMpxV/Indonesia/JK-NIHRD-MP276/2023 | EPI_ISL_18798841 | 197,196 | 109x |
| hMpxV/Indonesia/JK-NIHRD-MP279/2023 | EPI_ISL_18798842 | 197,196 | 148x |
| hMpxV/Indonesia/JK-Biokes-MP239/2023 | EPI_ISL_19159108 | 197,195 | 46x |
| hMpxV/Indonesia/JB-Biokes-MP294/2023 | EPI_ISL_19159109 | 197,192 | 29x |
| hMpxV/Indonesia/JB-Biokes-MP295/2023 | EPI_ISL_19159110 | 197,192 | 136x |
| hMpxV/Indonesia/JK-Biokes-MP302/2023 | EPI_ISL_19159111 | 197,184 | 23x |
| hMpxV/Indonesia/BT-Biokes-MP303/2023 | EPI_ISL_19159112 | 197,183 | 274x |
| hMpxV/Indonesia/JK-Biokes-MP310/2023 | EPI_ISL_19159113 | 197,182 | 198x |
| hMpxV/Indonesia/JK-Biokes-MP311/2023 | EPI_ISL_19159114 | 197,182 | 35x |
| hMpxV/Indonesia/JK-Biokes-MP010/2024 | EPI_ISL_19159115 | 197,181 | 439x |
| hMpxV/Indonesia/JK-Biokes-MP011/2024 | EPI_ISL_19159116 | 197,181 | 55x |
| hMpxV/Indonesia/JK-Biokes-MP016/2024 | EPI_ISL_19159117 | 197,177 | 17x |
| hMpxV/Indonesia/JK-Biokes-MP018/2024 | EPI_ISL_19159118 | 197,166 | 18x |
| hMpxV/Indonesia/JK-Biokes-MP020/2024 | EPI_ISL_19159119 | 197,163 | 4,071x |
| hMpxV/Indonesia/JK-Biokes-MP029/2024 | EPI_ISL_19159120 | 197,095 | 26x |

**Supplementary material 5.** The molecular signal analysis with TempEst, the Indonesian tree both with and without imputation of ambiguous sequences showed a very weak molecular clock signal


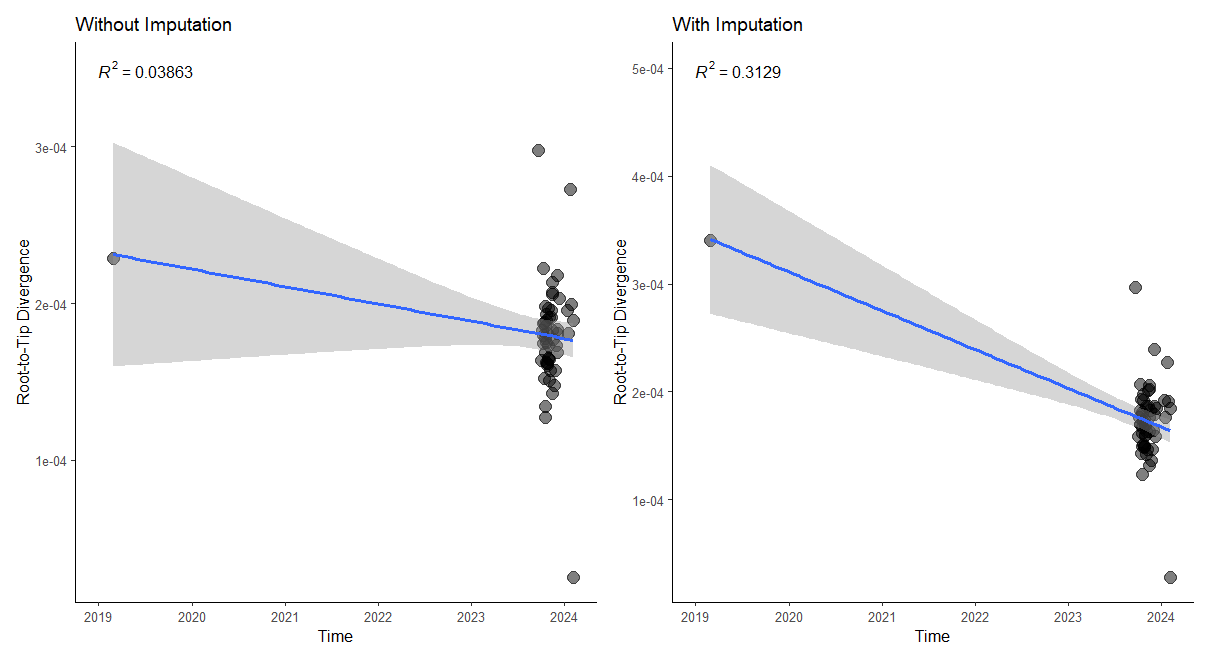


**Supplementary material 6.** The molecular signal analysis with TempEst, the ASEAN tree without imputation of ambiguous sequences showed better performance in molecular signal analysis than with imputation


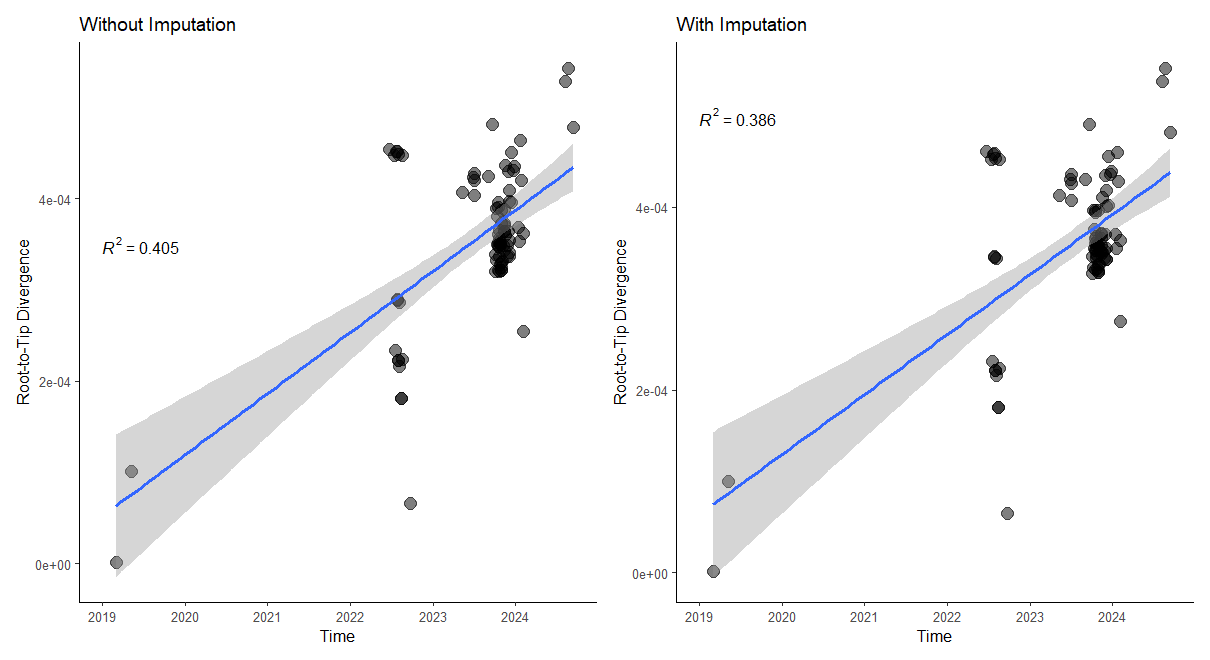


**Supplementary material 7.** The molecular signal analysis with TempEst showed the outliers in root-to-tip analysis and phylogenetic tree (red dots)


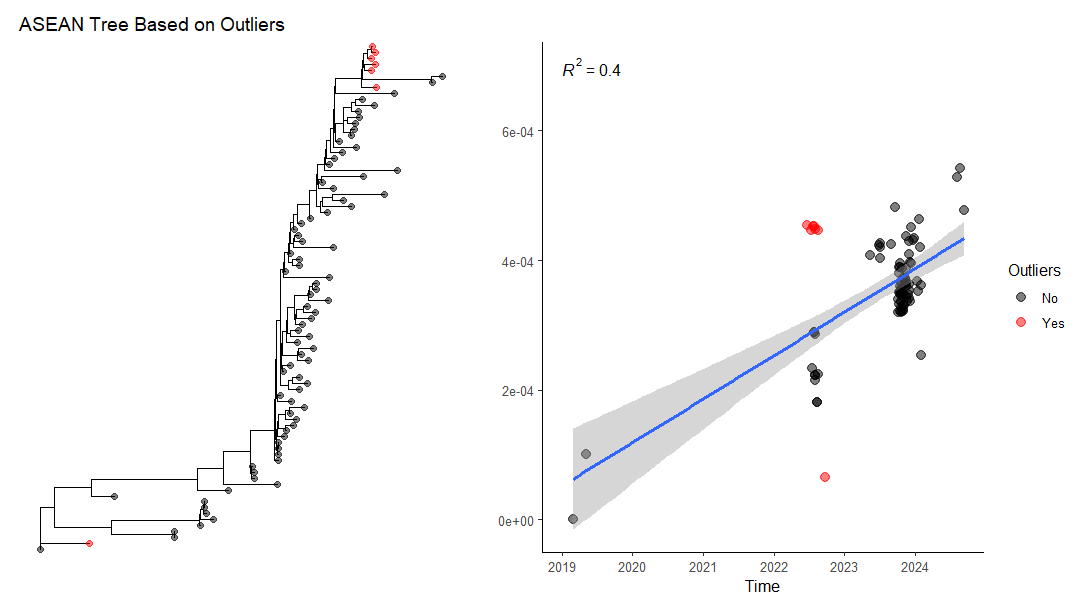


**Supplementary material 8.** The molecular signal analysis with TempEst of ASEAN tree after removing the outliers showed the strong molecular clock signal (R-square = 0.66)


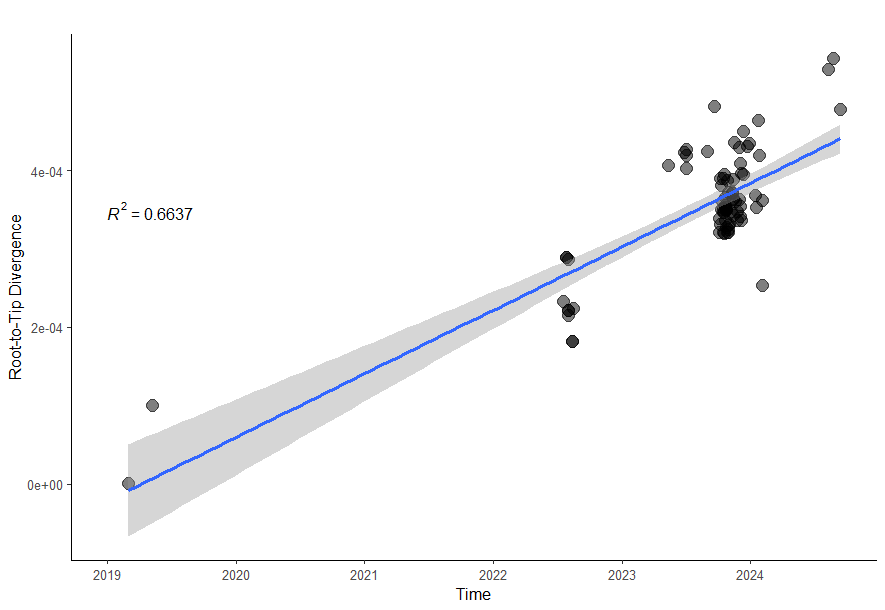


**Supplementary material 9.** The Treetime output of molecular clock analysis of ASEAN time-calibrated tree. The TMRCA and introduction time was estimated at 2018.5 (2018-07-02)


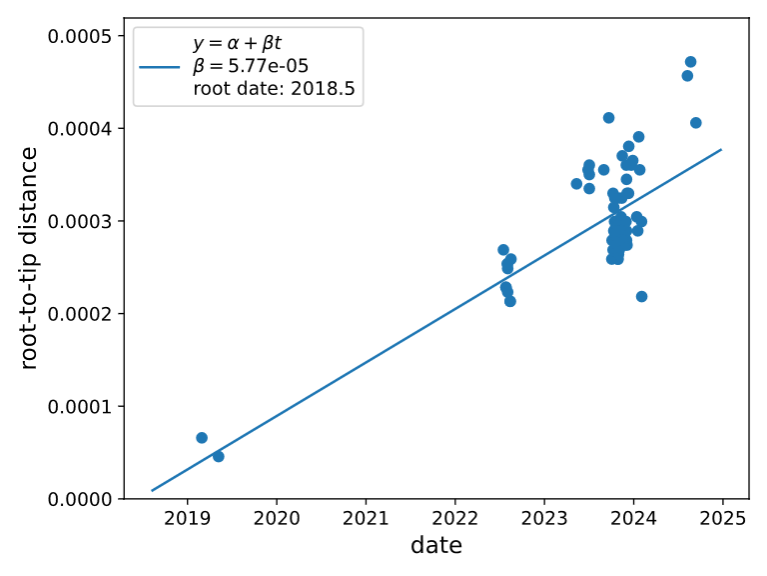


**Supplementary material 10.** List of GISAID and GenBank sequences that including in this study

| **Accession ID** | **Date Access** | **Submitting lab** | **Authors** | **Publications** |
| --- | --- | --- | --- | --- |
| NC_063383.1 | 2024-11-29 | - | Mauldin,M.R., McCollum,A.M., Nakazawa,Y.J. Mandra,A., Whitehouse,E.R., Davidson,W., Zhao,H., Gao,J., Li,Y., Doty,J., Yinka-Ogunleye,A., Akinpelu,A., Aruna,O., Naidoo,D., Lewandowski,K., Afrough,B., Graham,V., Aarons,E., Hewson,R., Vipond,R., Dunning,J., Chand,M., Brown,C., Cohen-Gihon,I., Erez,N., Shifman,O., Israeli,O., Sharon,M., Schwartz,E., Beth-Din,A., Zvi,A., Mak,T.M., Ng,Y.K., Cui,L., Lin,R.T.P., Olson,V.A., Brooks,T., Paran,N., Ihekweazu,C. and Reynolds,M.G. | Exportation of Monkeypox Virus From the African Continent (doi: 10.1093/infdis/jiaa559) |
| EPI_ISL_14011193 | 2024-11-29 | Thai Red Cross Emerging Infectious Diseases Clinical Center and Faculty of Medicine, | Kusak Kukiattikoon, Waritta Dararattanaroj, Rome Buathong, Supaporn  Wacharapluesadee, Sininat Petcharat, Ananporn Supataragul, Stefan Fernandez, Achawin  Rojanaviwat, Chonticha Klungthong, Pilailuk Okada, Khajohn Joonlasak, Chakkarat  Pitayawonganon, Opass Putcharoen | Phylogeny and molecular evolution of the first local monkeypox virus cluster in; Guangdong Province, China (https://doi.org/10.1038/s41467-023-44092-3); Multiple introductions of monkeypox virus to Ireland during the international mpox; outbreak, May 2022 to October 2023 (doi: 10.2807/1560-7917.ES.2024.29.16.2300505); Phylogenomic and Structural Analysis of the Monkeypox Virus Shows Evolution towards; Increased Stability (doi: 10.3390/v15010127 ) |
| EPI_ISL_14153982 | 2024-11-29 | National Institute of Health, Department of Medical Sciences, Ministry of Public Health, | Pilailuk Okada; Siripaporn Phuygun; Nuttida Thongpramul; Thanutsapa Thanadachakul; Kazuhisa Okada; Archawin Rojanawiwat; Chakkarat Pitayawonganon; Supakit Sirilak | Global genomic surveillance of monkeypox virus ( doi: 10.1038/s41591-024-03370-3 ); Multiple introductions of monkeypox virus to Ireland during the international mpox; outbreak, May 2022 to October 2023 (doi: 10.2807/1560-7917.ES.2024.29.16.2300505); Phylogenomic and Structural Analysis of the Monkeypox Virus Shows Evolution towards; Increased Stability (doi: 10.3390/v15010127 ); Genetic Features of 84 Genomes of Monkeypox Virus in Recent Circulation — Beijing; Municipality, China, 2023 (doi: 10.46234/ccdcw2023.173); Molecular Evolution of Protein Sequences and Codon Usage in Monkeypox Viruses (https://doi.org/10.1093/gpbjnl/qzad003); Evolutionary trajectory and characteristics of Mpox virus in 2023 based on a large-scale; genomic surveillance in Shenzhen, China (https://doi.org/10.1038/s41467-024-51737-4); Comparative genome analysis reveals driving forces behind Monkeypox virus evolution; and sheds light on the role of ATC trinucleotide motif (doi: 10.1093/ve/veae043 ); Complete Genome Sequence Analysis of the First Imported Mpox Virus Clade Ib Variant in China (doi: 10.3390/pathogens14010102); Structural insights into tecovirimat antiviral activity and poxvirus resistance (https://doi.org/10.1038/s41564-025-01936-6); Genomic epidemiology reveals 2022 mpox epidemic in New York City governed by heavy-; tailed sexual contact networks (doi: 10.1101/2024.07.30.24311083 ) |
| EPI_ISL_14721255 | 2024-11-29 | National Public Health Laboratory, National Centre for Infectious Diseases | Yichen Ding, Benny Yeo, Daniel Lim, Zhenyang Zhou, Royce Ang, Samuel Loo, Lin Cui, Raymond Tzer Pin Lin | Global genomic surveillance of monkeypox virus ( doi: 10.1038/s41591-024-03370-3 ); Retrospective detection of asymptomatic monkeypox virus infections among male sexual; health clinic attendees in Belgium (2022-08-12); Multiple introductions of monkeypox virus to Ireland during the international mpox; outbreak, May 2022 to October 2023 (doi: 10.2807/1560-7917.ES.2024.29.16.2300505); Phylogenomic and Structural Analysis of the Monkeypox Virus Shows Evolution towards; Increased Stability (doi: 10.3390/v15010127 ); Genetic Features of 84 Genomes of Monkeypox Virus in Recent Circulation — Beijing; Municipality, China, 2023 (doi: 10.46234/ccdcw2023.173); Molecular Evolution of Protein Sequences and Codon Usage in Monkeypox Viruses (https://doi.org/10.1093/gpbjnl/qzad003); Evolutionary trajectory and characteristics of Mpox virus in 2023 based on a large-scale; genomic surveillance in Shenzhen, China (https://doi.org/10.1038/s41467-024-51737-4); Comparative genome analysis reveals driving forces behind Monkeypox virus evolution; and sheds light on the role of ATC trinucleotide motif (doi: 10.1093/ve/veae043 ); Genomic epidemiology reveals 2022 mpox epidemic in New York City governed by heavy-; tailed sexual contact networks (doi: 10.1101/2024.07.30.24311083 ) |
| EPI_ISL_14721259 | 2024-11-29 | National Public Health Laboratory, National Centre for Infectious Diseases | Yichen Ding, Benny Yeo, Daniel Lim, Zhenyang Zhou, Royce Ang, Samuel Loo, Lin Cui, Raymond Tzer Pin Lin | Global genomic surveillance of monkeypox virus ( doi: 10.1038/s41591-024-03370-3 ); Multiple introductions of monkeypox virus to Ireland during the international mpox; outbreak, May 2022 to October 2023 (doi: 10.2807/1560-7917.ES.2024.29.16.2300505); Phylogenomic and Structural Analysis of the Monkeypox Virus Shows Evolution towards; Increased Stability (doi: 10.3390/v15010127 ); Molecular Evolution of Protein Sequences and Codon Usage in Monkeypox Viruses (https://doi.org/10.1093/gpbjnl/qzad003); Evolutionary trajectory and characteristics of Mpox virus in 2023 based on a large-scale; genomic surveillance in Shenzhen, China (https://doi.org/10.1038/s41467-024-51737-4); Comparative genome analysis reveals driving forces behind Monkeypox virus evolution; and sheds light on the role of ATC trinucleotide motif (doi: 10.1093/ve/veae043 ); Genomic epidemiology reveals 2022 mpox epidemic in New York City governed by heavy-; tailed sexual contact networks (doi: 10.1101/2024.07.30.24311083 ) |
| EPI_ISL_14721262 | 2024-11-29 | National Public Health Laboratory, National Centre for Infectious Diseases | Yichen Ding, Benny Yeo, Daniel Lim, Zhenyang Zhou, Royce Ang, Samuel Loo, Lin Cui, Raymond Tzer Pin Lin | Global genomic surveillance of monkeypox virus ( doi: 10.1038/s41591-024-03370-3 ); Multiple introductions of monkeypox virus to Ireland during the international mpox; outbreak, May 2022 to October 2023 (doi: 10.2807/1560-7917.ES.2024.29.16.2300505); Phylogenomic and Structural Analysis of the Monkeypox Virus Shows Evolution towards; Increased Stability (doi: 10.3390/v15010127 ); Genetic Features of 84 Genomes of Monkeypox Virus in Recent Circulation — Beijing; Municipality, China, 2023 (doi: 10.46234/ccdcw2023.173); Molecular Evolution of Protein Sequences and Codon Usage in Monkeypox Viruses (https://doi.org/10.1093/gpbjnl/qzad003); Evolutionary trajectory and characteristics of Mpox virus in 2023 based on a large-scale; genomic surveillance in Shenzhen, China (https://doi.org/10.1038/s41467-024-51737-4); Comparative genome analysis reveals driving forces behind Monkeypox virus evolution; and sheds light on the role of ATC trinucleotide motif (doi: 10.1093/ve/veae043 ); Genomic epidemiology reveals 2022 mpox epidemic in New York City governed by heavy-; tailed sexual contact networks (doi: 10.1101/2024.07.30.24311083 ) |
| EPI_ISL_14721265 | 2024-11-29 | National Public Health Laboratory, National Centre for Infectious Diseases | Yichen Ding, Benny Yeo, Daniel Lim, Zhenyang Zhou, Royce Ang, Samuel Loo, Lin Cui, Raymond Tzer Pin Lin | Global genomic surveillance of monkeypox virus ( doi: 10.1038/s41591-024-03370-3 ); Phylogeny and molecular evolution of the first local monkeypox virus cluster in; Guangdong Province, China (https://doi.org/10.1038/s41467-023-44092-3); Multiple introductions of monkeypox virus to Ireland during the international mpox; outbreak, May 2022 to October 2023 (doi: 10.2807/1560-7917.ES.2024.29.16.2300505); Phylogenomic and Structural Analysis of the Monkeypox Virus Shows Evolution towards; Increased Stability (doi: 10.3390/v15010127 ); Genetic Features of 84 Genomes of Monkeypox Virus in Recent Circulation — Beijing; Municipality, China, 2023 (doi: 10.46234/ccdcw2023.173); Molecular Evolution of Protein Sequences and Codon Usage in Monkeypox Viruses (https://doi.org/10.1093/gpbjnl/qzad003); Evolutionary trajectory and characteristics of Mpox virus in 2023 based on a large-scale; genomic surveillance in Shenzhen, China (https://doi.org/10.1038/s41467-024-51737-4); Comparative genome analysis reveals driving forces behind Monkeypox virus evolution; and sheds light on the role of ATC trinucleotide motif (doi: 10.1093/ve/veae043 ); Structural insights into tecovirimat antiviral activity and poxvirus resistance (https://doi.org/10.1038/s41564-025-01936-6); Genomic epidemiology reveals 2022 mpox epidemic in New York City governed by heavy-; tailed sexual contact networks (doi: 10.1101/2024.07.30.24311083 ) |
| EPI_ISL_15373792 | 2024-11-29 | Department of immunology and microbiology - Pasteur Institute in Ho Chi Minh city | Manh H. Dao, Nhung H. P. Vu, Hang T. T. Pham, Thang M. Cao, Thinh V. Nguyen, Quang | Global genomic surveillance of monkeypox virus ( doi: 10.1038/s41591-024-03370-3 ); Phylogeny and molecular evolution of the first local monkeypox virus cluster in; Guangdong Province, China (https://doi.org/10.1038/s41467-023-44092-3); Phylogenomic and Structural Analysis of the Monkeypox Virus Shows Evolution towards; Increased Stability (doi: 10.3390/v15010127 ); Genetic Features of 84 Genomes of Monkeypox Virus in Recent Circulation — Beijing; Municipality, China, 2023 (doi: 10.46234/ccdcw2023.173); Complete Genome Sequence Analysis of the First Imported Mpox Virus Clade Ib Variant in China (doi: 10.3390/pathogens14010102); Structural insights into tecovirimat antiviral activity and poxvirus resistance (https://doi.org/10.1038/s41564-025-01936-6); A comprehensive review of monkeypox virus and mpox characteristics (doi: 10.3389/fcimb.2024.1360586) |
| EPI_ISL_15380492 | 2024-11-29 | Molecular Biology Laboratory, Research Institute for Tropical Medicine | Samantha Louise P. Bado, Niquitta B. Galap, Bea C. Mateo, Chelsea Mae M. Reyes, Amalea Dulcene Nicolasora, Miguel Francisco B. Abulencia, Francisco Gerardo M. Polotan  on behalf of the Research Institute for Tropical Medicine | Global genomic surveillance of monkeypox virus ( doi: 10.1038/s41591-024-03370-3 ); Multiple introductions of monkeypox virus to Ireland during the international mpox; outbreak, May 2022 to October 2023 (doi: 10.2807/1560-7917.ES.2024.29.16.2300505); Phylogenomic and Structural Analysis of the Monkeypox Virus Shows Evolution towards; Increased Stability (doi: 10.3390/v15010127 ); Genetic Features of 84 Genomes of Monkeypox Virus in Recent Circulation — Beijing; Municipality, China, 2023 (doi: 10.46234/ccdcw2023.173); Update of the Genetic Variability of Monkeypox Virus Clade IIb Lineage B.1 (2024-09-11); Complete Genome Sequence Analysis of the First Imported Mpox Virus Clade Ib Variant in China (doi: 10.3390/pathogens14010102); Structural insights into tecovirimat antiviral activity and poxvirus resistance (https://doi.org/10.1038/s41564-025-01936-6) |
| EPI_ISL_17187497 | 2024-11-29 | Thai Red Cross Emerging Infectious Diseases Clinical Center and Faculty of Medicine, | Suppasit srisaeng, Praepoly Ruekmuang, Kusuma Swangpun, Arriya Panchaiyaphum, Pakita Salaeh, Natpusda Kongmaung, Pornsiri Limwattanawong, Noree Pholprasert,  Montriya Unteamsom, Kanjana Jeknok, Withak Withaksabut, Sunisa Nilda, Artorn Niakul,  Sopon Iamsirithaworn, Thitipong Yingyong, Rossaporn Kittiyaowamarn, Rome Buathong,  Ratanaporn Tangwangvivat, Supaporn Wacharapluesadee, Sininat Petcharat, Ananporn  Supataragul, Stefan Fernandez, Achawin Rojanaviwat, Chonticha Klungthong, Pilailuk  Okada, Khajohn Joonlasak, Chakkarat Pitayawonganon, Opass Putcharoen | Global genomic surveillance of monkeypox virus ( doi: 10.1038/s41591-024-03370-3 ); Multiple introductions of monkeypox virus to Ireland during the international mpox; outbreak, May 2022 to October 2023 (doi: 10.2807/1560-7917.ES.2024.29.16.2300505); Complete Genome Sequence Analysis of the First Imported Mpox Virus Clade Ib Variant in China (doi: 10.3390/pathogens14010102) |
| EPI_ISL_17187498 | 2024-11-29 | Thai Red Cross Emerging Infectious Diseases Clinical Center and Faculty of Medicine, | Suppasit srisaeng, Praepoly Ruekmuang, Kusuma Swangpun, Arriya Panchaiyaphum, Pakita Salaeh, Natpusda Kongmaung, Pornsiri Limwattanawong, Noree Pholprasert,  Montriya Unteamsom, Kanjana Jeknok, Withak Withaksabut, Sunisa Nilda, Artorn Niakul,  Sopon Iamsirithaworn, Thitipong Yingyong, Rossaporn Kittiyaowamarn, Rome Buathong,  Ratanaporn Tangwangvivat, Supaporn Wacharapluesadee, Sininat Petcharat, Ananporn  Supataragul, Stefan Fernandez, Achawin Rojanaviwat, Chonticha Klungthong, Pilailuk  Okada, Khajohn Joonlasak, Chakkarat Pitayawonganon, Opass Putcharoen | Global genomic surveillance of monkeypox virus ( doi: 10.1038/s41591-024-03370-3 ); Multiple introductions of monkeypox virus to Ireland during the international mpox; outbreak, May 2022 to October 2023 (doi: 10.2807/1560-7917.ES.2024.29.16.2300505) |
| EPI_ISL_17187499 | 2024-11-29 | Thai Red Cross Emerging Infectious Diseases Clinical Center and Faculty of Medicine, | Suppasit srisaeng, Praepoly Ruekmuang, Kusuma Swangpun, Arriya Panchaiyaphum, Pakita Salaeh, Natpusda Kongmaung, Pornsiri Limwattanawong, Noree Pholprasert,  Montriya Unteamsom, Kanjana Jeknok, Withak Withaksabut, Sunisa Nilda, Artorn Niakul,  Sopon Iamsirithaworn, Thitipong Yingyong, Rossaporn Kittiyaowamarn, Rome Buathong,  Ratanaporn Tangwangvivat, Supaporn Wacharapluesadee, Sininat Petcharat, Ananporn  Supataragul, Stefan Fernandez, Achawin Rojanaviwat, Chonticha Klungthong, Pilailuk  Okada, Khajohn Joonlasak, Chakkarat Pitayawonganon, Opass Putcharoen | Global genomic surveillance of monkeypox virus ( doi: 10.1038/s41591-024-03370-3 ); Multiple introductions of monkeypox virus to Ireland during the international mpox; outbreak, May 2022 to October 2023 (doi: 10.2807/1560-7917.ES.2024.29.16.2300505) |
| EPI_ISL_17187500 | 2024-11-29 | Thai Red Cross Emerging Infectious Diseases Clinical Center and Faculty of Medicine, | Nungrathai Srisong, Praepoly Ruekmuang, Kusuma Swangpun, Arriya Panchaiyaphum, Pakita Salaeh, Natpusda Kongmaung, Pornsiri Limwattanawong, Noree Pholprasert,  Montriya Unteamsom, Kanjana Jeknok, Withak Withaksabut, Sunisa Nilda, Artorn Niakul,  Sopon Iamsirithaworn, Thitipong Yingyong, Rossaporn Kittiyaowamarn, Rome Buathong,  Ratanaporn Tangwangvivat, Supaporn Wacharapluesadee, Sininat Petcharat, Ananporn  Supataragul, Stefan Fernandez, Achawin Rojanaviwat, Chonticha Klungthong, Pilailuk  Okada, Khajohn Joonlasak, Chakkarat Pitayawonganon, Opass Putcharoen | Global genomic surveillance of monkeypox virus ( doi: 10.1038/s41591-024-03370-3 ); Phylogeny and molecular evolution of the first local monkeypox virus cluster in; Guangdong Province, China (https://doi.org/10.1038/s41467-023-44092-3); Multiple introductions of monkeypox virus to Ireland during the international mpox; outbreak, May 2022 to October 2023 (doi: 10.2807/1560-7917.ES.2024.29.16.2300505); Evolutionary trajectory and characteristics of Mpox virus in 2023 based on a large-scale; genomic surveillance in Shenzhen, China (https://doi.org/10.1038/s41467-024-51737-4) |
| EPI_ISL_17187501 | 2024-11-29 | Thai Red Cross Emerging Infectious Diseases Clinical Center and Faculty of Medicine, | Nungrathai Srisong, Praepoly Ruekmuang, Kusuma Swangpun, Arriya Panchaiyaphum, Pakita Salaeh, Natpusda Kongmaung, Pornsiri Limwattanawong, Noree Pholprasert,  Montriya Unteamsom, Kanjana Jeknok, Withak Withaksabut, Sunisa Nilda, Artorn Niakul,  Sopon Iamsirithaworn, Thitipong Yingyong, Rossaporn Kittiyaowamarn, Rome Buathong,  Ratanaporn Tangwangvivat, Supaporn Wacharapluesadee, Sininat Petcharat, Ananporn  Supataragul, Stefan Fernandez, Achawin Rojanaviwat, Chonticha Klungthong, Pilailuk  Okada, Khajohn Joonlasak, Chakkarat Pitayawonganon, Opass Putcharoen | Phylogeny and molecular evolution of the first local monkeypox virus cluster in; Guangdong Province, China (https://doi.org/10.1038/s41467-023-44092-3); Multiple introductions of monkeypox virus to Ireland during the international mpox; outbreak, May 2022 to October 2023 (doi: 10.2807/1560-7917.ES.2024.29.16.2300505); Evolutionary trajectory and characteristics of Mpox virus in 2023 based on a large-scale; genomic surveillance in Shenzhen, China (https://doi.org/10.1038/s41467-024-51737-4) |
| EPI_ISL_17187502 | 2024-11-29 | Thai Red Cross Emerging Infectious Diseases Clinical Center and Faculty of Medicine, | Supanut Chotichavalrattanakul, , Praepoly Ruekmuang, Kusuma Swangpun, Arriya Panchaiyaphum, Pakita Salaeh, Natpusda Kongmaung, Pornsiri Limwattanawong, Noree Pholprasert,  Montriya Unteamsom, Kanjana Jeknok, Withak Withaksabut, Sunisa Nilda, Artorn Niakul,  Sopon Iamsirithaworn, Thitipong Yingyong, Rossaporn Kittiyaowamarn, Rome Buathong,  Ratanaporn Tangwangvivat, Supaporn Wacharapluesadee, Sininat Petcharat, Ananporn  Supataragul, Stefan Fernandez, Achawin Rojanaviwat, Chonticha Klungthong, Pilailuk  Okada, Khajohn Joonlasak, Chakkarat Pitayawonganon, Opass Putcharoen | Global genomic surveillance of monkeypox virus ( doi: 10.1038/s41591-024-03370-3 ); Phylogeny and molecular evolution of the first local monkeypox virus cluster in; Guangdong Province, China (https://doi.org/10.1038/s41467-023-44092-3); Multiple introductions of monkeypox virus to Ireland during the international mpox; outbreak, May 2022 to October 2023 (doi: 10.2807/1560-7917.ES.2024.29.16.2300505); Evolutionary trajectory and characteristics of Mpox virus in 2023 based on a large-scale; genomic surveillance in Shenzhen, China (https://doi.org/10.1038/s41467-024-51737-4) |
| EPI_ISL_17187503 | 2024-11-29 | Thai Red Cross Emerging Infectious Diseases Clinical Center and Faculty of Medicine, | Phawinee Montri, Praepoly Ruekmuang, Kusuma Swangpun, Arriya Panchaiyaphum, Pakita Salaeh, Natpusda Kongmaung, Pornsiri Limwattanawong, Noree Pholprasert,  Montriya Unteamsom, Kanjana Jeknok, Withak Withaksabut, Sunisa Nilda, Artorn Niakul,  Sopon Iamsirithaworn, Thitipong Yingyong, Rossaporn Kittiyaowamarn, Rome Buathong,  Ratanaporn Tangwangvivat, Supaporn Wacharapluesadee, Sininat Petcharat, Ananporn  Supataragul, Stefan Fernandez, Achawin Rojanaviwat, Chonticha Klungthong, Pilailuk  Okada, Khajohn Joonlasak, Chakkarat Pitayawonganon, Opass Putcharoen | Phylogeny and molecular evolution of the first local monkeypox virus cluster in; Guangdong Province, China (https://doi.org/10.1038/s41467-023-44092-3); Multiple introductions of monkeypox virus to Ireland during the international mpox; outbreak, May 2022 to October 2023 (doi: 10.2807/1560-7917.ES.2024.29.16.2300505); Evolutionary trajectory and characteristics of Mpox virus in 2023 based on a large-scale; genomic surveillance in Shenzhen, China (https://doi.org/10.1038/s41467-024-51737-4) |
| EPI_ISL_17187504 | 2024-11-29 | Thai Red Cross Emerging Infectious Diseases Clinical Center and Faculty of Medicine, | Phawinee Montri, Praepoly Ruekmuang, Kusuma Swangpun, Arriya Panchaiyaphum, Pakita Salaeh, Natpusda Kongmaung, Pornsiri Limwattanawong, Noree Pholprasert,  Montriya Unteamsom, Kanjana Jeknok, Withak Withaksabut, Sunisa Nilda, Artorn Niakul,  Sopon Iamsirithaworn, Thitipong Yingyong, Rossaporn Kittiyaowamarn, Rome Buathong,  Ratanaporn Tangwangvivat, Supaporn Wacharapluesadee, Sininat Petcharat, Ananporn  Supataragul, Stefan Fernandez, Achawin Rojanaviwat, Chonticha Klungthong, Pilailuk  Okada, Khajohn Joonlasak, Chakkarat Pitayawonganon, Opass Putcharoen | Global genomic surveillance of monkeypox virus ( doi: 10.1038/s41591-024-03370-3 ); Multiple introductions of monkeypox virus to Ireland during the international mpox; outbreak, May 2022 to October 2023 (doi: 10.2807/1560-7917.ES.2024.29.16.2300505) |
| EPI_ISL_17187505 | 2024-11-29 | Thai Red Cross Emerging Infectious Diseases Clinical Center and Faculty of Medicine, | Nungrathai Srisong, Praepoly Ruekmuang, Kusuma Swangpun, Arriya Panchaiyaphum, Pakita Salaeh, Natpusda Kongmaung, Pornsiri Limwattanawong, Noree Pholprasert,  Montriya Unteamsom, Kanjana Jeknok, Withak Withaksabut, Sunisa Nilda, Artorn Niakul,  Sopon Iamsirithaworn, Thitipong Yingyong, Rossaporn Kittiyaowamarn, Rome Buathong,  Ratanaporn Tangwangvivat, Supaporn Wacharapluesadee, Sininat Petcharat, Ananporn  Supataragul, Stefan Fernandez, Achawin Rojanaviwat, Chonticha Klungthong, Pilailuk  Okada, Khajohn Joonlasak, Chakkarat Pitayawonganon, Opass Putcharoen | Phylogeny and molecular evolution of the first local monkeypox virus cluster in; Guangdong Province, China (https://doi.org/10.1038/s41467-023-44092-3); Multiple introductions of monkeypox virus to Ireland during the international mpox; outbreak, May 2022 to October 2023 (doi: 10.2807/1560-7917.ES.2024.29.16.2300505) |
| EPI_ISL_18436040 | 2024-11-29 | National Institute of Health Research and Development | Fajar Nur Sulistyahadi, Arie Ardiansyah Nugraha, Hana Apsari Pawestri, Kartika Dewi Puspa, Herna, Subangkit, IGM Wirabrata | Complete Genome Sequence Analysis of the First Imported Mpox Virus Clade Ib Variant in China (doi: 10.3390/pathogens14010102); Structural insights into tecovirimat antiviral activity and poxvirus resistance (https://doi.org/10.1038/s41564-025-01936-6); |
| EPI_ISL_18436041 | 2024-11-29 | National Institute of Health Research and Development | Fajar Nur Sulistyahadi, Arie Ardiansyah Nugraha, Hana Apsari Pawestri, Kartika Dewi Puspa, Herna, Subangkit, IGM Wirabrata | Global genomic surveillance of monkeypox virus ( doi: 10.1038/s41591-024-03370-3 ); Evolutionary variation of the monkeypox virus detected for the first time in Nantong,; Jiangsu (2024-12-23); Complete Genome Sequence Analysis of the First Imported Mpox Virus Clade Ib Variant in China (doi: 10.3390/pathogens14010102); Structural insights into tecovirimat antiviral activity and poxvirus resistance (https://doi.org/10.1038/s41564-025-01936-6) |
| EPI_ISL_18463158 | 2024-11-29 | National Institute of Health Research and Development | Hana Apsari Pawestri, Arie Ardiansyah Nugraha, Fajar Nur Sulistyahadi, Hartanti Dian Ikawati, Kartika Dewi Puspa, Markus Evan Anggia, Subangkit, Nelis Imaningsih, IGM  Wirabrata | Global genomic surveillance of monkeypox virus ( doi: 10.1038/s41591-024-03370-3 ); Multiple introductions of monkeypox virus to Ireland during the international mpox; outbreak, May 2022 to October 2023 (doi: 10.2807/1560-7917.ES.2024.29.16.2300505); Evolutionary trajectory and characteristics of Mpox virus in 2023 based on a large-scale; genomic surveillance in Shenzhen, China (https://doi.org/10.1038/s41467-024-51737-4); Complete Genome Sequence Analysis of the First Imported Mpox Virus Clade Ib Variant in China (doi: 10.3390/pathogens14010102) |
| EPI_ISL_18463159 | 2024-11-29 | National Institute of Health Research and Development | Hana Apsari Pawestri, Arie Ardiansyah Nugraha, Fajar Nur Sulistyahadi, Hartanti Dian Ikawati, Kartika Dewi Puspa, Markus Evan Anggia, Subangkit, Nelis Imaningsih, IGM  Wirabrata | Global genomic surveillance of monkeypox virus ( doi: 10.1038/s41591-024-03370-3 ); Multiple introductions of monkeypox virus to Ireland during the international mpox; outbreak, May 2022 to October 2023 (doi: 10.2807/1560-7917.ES.2024.29.16.2300505); Evolutionary trajectory and characteristics of Mpox virus in 2023 based on a large-scale; genomic surveillance in Shenzhen, China (https://doi.org/10.1038/s41467-024-51737-4) |
| EPI_ISL_18463160 | 2024-11-29 | National Institute of Health Research and Development | Hana Apsari Pawestri, Arie Ardiansyah Nugraha, Fajar Nur Sulistyahadi, Hartanti Dian Ikawati, Kartika Dewi Puspa, Markus Evan Anggia, Subangkit, Nelis Imaningsih, IGM  Wirabrata | Global genomic surveillance of monkeypox virus ( doi: 10.1038/s41591-024-03370-3 ); Multiple introductions of monkeypox virus to Ireland during the international mpox; outbreak, May 2022 to October 2023 (doi: 10.2807/1560-7917.ES.2024.29.16.2300505); Evolutionary trajectory and characteristics of Mpox virus in 2023 based on a large-scale; genomic surveillance in Shenzhen, China (https://doi.org/10.1038/s41467-024-51737-4); Complete Genome Sequence Analysis of the First Imported Mpox Virus Clade Ib Variant in China (doi: 10.3390/pathogens14010102) |
| EPI_ISL_18463161 | 2024-11-29 | National Institute of Health Research and Development | Hana Apsari Pawestri, Arie Ardiansyah Nugraha, Fajar Nur Sulistyahadi, Hartanti Dian Ikawati, Kartika Dewi Puspa, Markus Evan Anggia, Subangkit, Nelis Imaningsih, IGM  Wirabrata | Global genomic surveillance of monkeypox virus ( doi: 10.1038/s41591-024-03370-3 ); Multiple introductions of monkeypox virus to Ireland during the international mpox; outbreak, May 2022 to October 2023 (doi: 10.2807/1560-7917.ES.2024.29.16.2300505) |
| EPI_ISL_18467794 | 2024-11-29 | National Institute of Health Research and Development | Fajar Nur Sulistyahadi, Hana Apsari Pawestri, Arie Ardiansyah Nugraha, Hartanti Dian Ikawati, Kartika Dewi Puspa, Subangkit, IGM Wirabrata | Global genomic surveillance of monkeypox virus ( doi: 10.1038/s41591-024-03370-3 ); Complete Genome Sequence Analysis of the First Imported Mpox Virus Clade Ib Variant in China (doi: 10.3390/pathogens14010102) |
| EPI_ISL_18467795 | 2024-11-29 | National Institute of Health Research and Development | Hana Apsari Pawestri, Arie Ardiansyah Nugraha, Fajar Nur Sulistyahadi, Hartanti Dian  Ikawati, Kartika Dewi Puspa, Subangkit, IGM Wirabrata | Global genomic surveillance of monkeypox virus ( doi: 10.1038/s41591-024-03370-3 ) |
| EPI_ISL_18467796 | 2024-11-29 | National Institute of Health Research and Development | Hana Apsari Pawestri, Arie Ardiansyah Nugraha, Fajar Nur Sulistyahadi, Hartanti Dian  Ikawati, Kartika Dewi Puspa, Subangkit, IGM Wirabrata | Global genomic surveillance of monkeypox virus ( doi: 10.1038/s41591-024-03370-3 ) |
| EPI_ISL_18467797 | 2024-11-29 | National Institute of Health Research and Development | Arie Ardiansyah Nugraha, Fajar Nur Sulistyahadi, Hartanti Dian Ikawati, Kartika Dewi  Puspa, Hana Apsari Pawestri, Subangkit, IGM Wirabrata | Global genomic surveillance of monkeypox virus ( doi: 10.1038/s41591-024-03370-3 ) |
| EPI_ISL_18467798 | 2024-11-29 | National Institute of Health Research and Development | Fajar Nur Sulistyahadi, Hana Apsari Pawestri, Arie Ardiansyah Nugraha, Hartanti Dian Ikawati, Kartika Dewi Puspa, Subangkit, IGM Wirabrata | Global genomic surveillance of monkeypox virus ( doi: 10.1038/s41591-024-03370-3 ); Structural insights into tecovirimat antiviral activity and poxvirus resistance (https://doi.org/10.1038/s41564-025-01936-6) |
| EPI_ISL_18467799 | 2024-11-29 | National Institute of Health Research and Development | Hana Apsari Pawestri, Arie Ardiansyah Nugraha, Fajar Nur Sulistyahadi, Hartanti Dian Ikawati, Kartika Dewi Puspa, Markus Evan Anggia, Subangkit, Nelis Imaningsih, IGM  Wirabrata | Global genomic surveillance of monkeypox virus ( doi: 10.1038/s41591-024-03370-3 ) |
| EPI_ISL_18467800 | 2024-11-29 | National Institute of Health Research and Development | Arie Ardiansyah Nugraha, Fajar Nur Sulistyahadi, Hartanti Dian Ikawati, Kartika Dewi Puspa, Hana Apsari Pawestri, Subangkit, IGM Wirabrata | Global genomic surveillance of monkeypox virus ( doi: 10.1038/s41591-024-03370-3 ) |
| EPI_ISL_18467801 | 2024-11-29 | National Institute of Health Research and Development | Arie Ardiansyah Nugraha, Fajar Nur Sulistyahadi, Hartanti Dian Ikawati, Kartika Dewi Puspa, Hana Apsari Pawestri, Subangkit, IGM Wirabrata | Global genomic surveillance of monkeypox virus ( doi: 10.1038/s41591-024-03370-3 ) |
| EPI_ISL_18467802 | 2024-11-29 | National Institute of Health Research and Development | Fajar Nur Sulistyahadi, Hana Apsari Pawestri, Arie Ardiansyah Nugraha, Hartanti Dian Ikawati, Kartika Dewi Puspa, Subangkit, IGM Wirabrata | Global genomic surveillance of monkeypox virus ( doi: 10.1038/s41591-024-03370-3 ) |
| EPI_ISL_18467803 | 2024-11-29 | National Institute of Health Research and Development | Fajar Nur Sulistyahadi, Hana Apsari Pawestri, Arie Ardiansyah Nugraha, Hartanti Dian Ikawati, Kartika Dewi Puspa, Subangkit, IGM Wirabrata | Global genomic surveillance of monkeypox virus ( doi: 10.1038/s41591-024-03370-3 ); Structural insights into tecovirimat antiviral activity and poxvirus resistance (https://doi.org/10.1038/s41564-025-01936-6) |
| EPI_ISL_18467804 | 2024-11-29 | National Institute of Health Research and Development | Hana Apsari Pawestri, Arie Ardiansyah Nugraha, Fajar Nur Sulistyahadi, Hartanti Dian Ikawati, Kartika Dewi Puspa, Subangkit, IGM Wirabrata | Global genomic surveillance of monkeypox virus ( doi: 10.1038/s41591-024-03370-3 ) |
| EPI_ISL_18467805 | 2024-11-29 | National Institute of Health Research and Development | Arie Ardiansyah Nugraha, Fajar Nur Sulistyahadi, Hartanti Dian Ikawati, Kartika Dewi Puspa, Hana Apsari Pawestri, Subangkit, IGM Wirabrata | Global genomic surveillance of monkeypox virus ( doi: 10.1038/s41591-024-03370-3 ) |
| EPI_ISL_18467806 | 2024-11-29 | National Institute of Health Research and Development | Fajar Nur Sulistyahadi, Hana Apsari Pawestri, Arie Ardiansyah Nugraha, Hartanti Dian Ikawati, Kartika Dewi Puspa, Subangkit, IGM Wirabrata | Global genomic surveillance of monkeypox virus ( doi: 10.1038/s41591-024-03370-3 ); Complete Genome Sequence Analysis of the First Imported Mpox Virus Clade Ib Variant in China (doi: 10.3390/pathogens14010102) |
| EPI_ISL_18467807 | 2024-11-29 | National Institute of Health Research and Development | Fajar Nur Sulistyahadi, Hana Apsari Pawestri, Arie Ardiansyah Nugraha, Hartanti Dian Ikawati, Kartika Dewi Puspa, Subangkit, IGM Wirabrata | Global genomic surveillance of monkeypox virus ( doi: 10.1038/s41591-024-03370-3 ); |
| EPI_ISL_18467808 | 2024-11-29 | National Institute of Health Research and Development | Arie Ardiansyah Nugraha, Fajar Nur Sulistyahadi, Hartanti Dian Ikawati, Kartika Dewi Puspa, Hana Apsari Pawestri, Subangkit, IGM Wirabrata | Global genomic surveillance of monkeypox virus ( doi: 10.1038/s41591-024-03370-3 ); Complete Genome Sequence Analysis of the First Imported Mpox Virus Clade Ib Variant in China (doi: 10.3390/pathogens14010102) |
| EPI_ISL_18642356 | 2024-11-29 | National Institute of Health Research and Development | Hana Apsari Pawestri, Arie Ardiansyah Nugraha, Fajar Nur Sulistyahadi, Markus Evan Anggia, Subangkit, Herna, IGM Wirabrata | Global genomic surveillance of monkeypox virus ( doi: 10.1038/s41591-024-03370-3 ); Multiple introductions of monkeypox virus to Ireland during the international mpox; outbreak, May 2022 to October 2023 (doi: 10.2807/1560-7917.ES.2024.29.16.2300505) |
| EPI_ISL_18642357 | 2024-11-29 | National Institute of Health Research and Development | Hana Apsari Pawestri, Arie Ardiansyah Nugraha, Fajar Nur Sulistyahadi, Markus Evan Anggia, Subangkit, Herna, IGM Wirabrata | Global genomic surveillance of monkeypox virus ( doi: 10.1038/s41591-024-03370-3 ); Multiple introductions of monkeypox virus to Ireland during the international mpox; outbreak, May 2022 to October 2023 (doi: 10.2807/1560-7917.ES.2024.29.16.2300505) |
| EPI_ISL_18642358 | 2024-11-29 | National Institute of Health Research and Development | Hana Apsari Pawestri, Arie Ardiansyah Nugraha, Fajar Nur Sulistyahadi, Markus Evan Anggia, Subangkit, Herna, IGM Wirabrata | Global genomic surveillance of monkeypox virus ( doi: 10.1038/s41591-024-03370-3 ); Multiple introductions of monkeypox virus to Ireland during the international mpox; outbreak, May 2022 to October 2023 (doi: 10.2807/1560-7917.ES.2024.29.16.2300505) |
| EPI_ISL_18642359 | 2024-11-29 | National Institute of Health Research and Development | Hana Apsari Pawestri, Arie Ardiansyah Nugraha, Fajar Nur Sulistyahadi, Markus Evan Anggia, Subangkit, Herna, IGM Wirabrata | Global genomic surveillance of monkeypox virus ( doi: 10.1038/s41591-024-03370-3 ); Multiple introductions of monkeypox virus to Ireland during the international mpox; outbreak, May 2022 to October 2023 (doi: 10.2807/1560-7917.ES.2024.29.16.2300505); Complete Genome Sequence Analysis of the First Imported Mpox Virus Clade Ib Variant in China (doi: 10.3390/pathogens14010102) |
| EPI_ISL_18642360 | 2024-11-29 | National Institute of Health Research and Development | Hana Apsari Pawestri, Arie Ardiansyah Nugraha, Fajar Nur Sulistyahadi, Markus Evan Anggia, Subangkit, Herna, IGM Wirabrata | Global genomic surveillance of monkeypox virus ( doi: 10.1038/s41591-024-03370-3 ); Multiple introductions of monkeypox virus to Ireland during the international mpox; outbreak, May 2022 to October 2023 (doi: 10.2807/1560-7917.ES.2024.29.16.2300505) |
| EPI_ISL_18642361 | 2024-11-29 | National Institute of Health Research and Development | Hana Apsari Pawestri, Arie Ardiansyah Nugraha, Fajar Nur Sulistyahadi, Markus Evan Anggia, Subangkit, Herna, IGM Wirabrata | Global genomic surveillance of monkeypox virus ( doi: 10.1038/s41591-024-03370-3 ); Multiple introductions of monkeypox virus to Ireland during the international mpox; outbreak, May 2022 to October 2023 (doi: 10.2807/1560-7917.ES.2024.29.16.2300505) |
| EPI_ISL_18642362 | 2024-11-29 | National Institute of Health Research and Development | Hana Apsari Pawestri, Arie Ardiansyah Nugraha, Fajar Nur Sulistyahadi, Markus Evan Anggia, Subangkit, Herna, IGM Wirabrata | Global genomic surveillance of monkeypox virus ( doi: 10.1038/s41591-024-03370-3 ); Multiple introductions of monkeypox virus to Ireland during the international mpox; outbreak, May 2022 to October 2023 (doi: 10.2807/1560-7917.ES.2024.29.16.2300505); Complete Genome Sequence Analysis of the First Imported Mpox Virus Clade Ib Variant in China (doi: 10.3390/pathogens14010102) |
| EPI_ISL_18642363 | 2024-11-29 | National Institute of Health Research and Development | Hana Apsari Pawestri, Arie Ardiansyah Nugraha, Fajar Nur Sulistyahadi, Markus Evan Anggia, Subangkit, Herna, IGM Wirabrata | Global genomic surveillance of monkeypox virus ( doi: 10.1038/s41591-024-03370-3 ); Multiple introductions of monkeypox virus to Ireland during the international mpox; outbreak, May 2022 to October 2023 (doi: 10.2807/1560-7917.ES.2024.29.16.2300505) |
| EPI_ISL_18642364 | 2024-11-29 | National Institute of Health Research and Development | Hana Apsari Pawestri, Arie Ardiansyah Nugraha, Fajar Nur Sulistyahadi, Markus Evan Anggia, Subangkit, Herna, IGM Wirabrata | Global genomic surveillance of monkeypox virus ( doi: 10.1038/s41591-024-03370-3 ); Evolutionary variation of the monkeypox virus detected for the first time in Nantong,; Jiangsu (2024-12-23); Multiple introductions of monkeypox virus to Ireland during the international mpox; outbreak, May 2022 to October 2023 (doi: 10.2807/1560-7917.ES.2024.29.16.2300505) |
| EPI_ISL_18642365 | 2024-11-29 | National Institute of Health Research and Development | Hana Apsari Pawestri, Arie Ardiansyah Nugraha, Fajar Nur Sulistyahadi, Markus Evan Anggia, Subangkit, Herna, IGM Wirabrata | Global genomic surveillance of monkeypox virus ( doi: 10.1038/s41591-024-03370-3 ); Multiple introductions of monkeypox virus to Ireland during the international mpox; outbreak, May 2022 to October 2023 (doi: 10.2807/1560-7917.ES.2024.29.16.2300505) |
| EPI_ISL_18798834 | 2024-11-29 | National Institute of Health Research and Development | Hana Apsari Pawestri, Arie Ardiansyah Nugraha, Fajar Nur Sulistyahadi, Markus Evan  Anggia, Subangkit | Global genomic surveillance of monkeypox virus ( doi: 10.1038/s41591-024-03370-3 ) |
| EPI_ISL_18798835 | 2024-11-29 | National Institute of Health Research and Development | Hana Apsari Pawestri, Arie Ardiansyah Nugraha, Fajar Nur Sulistyahadi, Markus Evan  Anggia, Subangkit | Global genomic surveillance of monkeypox virus ( doi: 10.1038/s41591-024-03370-3 ) |
| EPI_ISL_18798836 | 2024-11-29 | National Institute of Health Research and Development | Hana Apsari Pawestri, Arie Ardiansyah Nugraha, Fajar Nur Sulistyahadi, Markus Evan  Anggia, Subangkit | Global genomic surveillance of monkeypox virus ( doi: 10.1038/s41591-024-03370-3 ) |
| EPI_ISL_18798837 | 2024-11-29 | National Institute of Health Research and Development | Hana Apsari Pawestri, Arie Ardiansyah Nugraha, Fajar Nur Sulistyahadi, Markus Evan  Anggia, Subangkit | Global genomic surveillance of monkeypox virus ( doi: 10.1038/s41591-024-03370-3 ) |
| EPI_ISL_18798838 | 2024-11-29 | National Institute of Health Research and Development | Hana Apsari Pawestri, Arie Ardiansyah Nugraha, Fajar Nur Sulistyahadi, Markus Evan  Anggia, Subangkit | Global genomic surveillance of monkeypox virus ( doi: 10.1038/s41591-024-03370-3 ) |
| EPI_ISL_18798839 | 2024-11-29 | National Institute of Health Research and Development | Hana Apsari Pawestri, Arie Ardiansyah Nugraha, Fajar Nur Sulistyahadi, Markus Evan  Anggia, Subangkit | Global genomic surveillance of monkeypox virus ( doi: 10.1038/s41591-024-03370-3 ); Complete Genome Sequence Analysis of the First Imported Mpox Virus Clade Ib Variant in China (doi: 10.3390/pathogens14010102) |
| EPI_ISL_18798840 | 2024-11-29 | National Institute of Health Research and Development | Hana Apsari Pawestri, Arie Ardiansyah Nugraha, Fajar Nur Sulistyahadi, Markus Evan  Anggia, Subangkit | Global genomic surveillance of monkeypox virus ( doi: 10.1038/s41591-024-03370-3 ) |
| EPI_ISL_18798841 | 2024-11-29 | National Institute of Health Research and Development | Hana Apsari Pawestri, Arie Ardiansyah Nugraha, Fajar Nur Sulistyahadi, Markus Evan  Anggia, Subangkit | Global genomic surveillance of monkeypox virus ( doi: 10.1038/s41591-024-03370-3 ) |
| EPI_ISL_18798842 | 2024-11-29 | National Institute of Health Research and Development | Hana Apsari Pawestri, Arie Ardiansyah Nugraha, Fajar Nur Sulistyahadi, Markus Evan  Anggia, Subangkit | Global genomic surveillance of monkeypox virus ( doi: 10.1038/s41591-024-03370-3 ); |
| EPI_ISL_19159108 | 2024-11-29 | Balai Besar Laboratorium Biologi Kesehatan | Hana Apsari Pawestri, Arie Ardiansyah Nugraha, Fajar Nur Sulistyahadi, Markus Evan  Anggia, Subangkit | Structural insights into tecovirimat antiviral activity and poxvirus resistance (https://doi.org/10.1038/s41564-025-01936-6); |
| EPI_ISL_19159109 | 2024-11-29 | Balai Besar Laboratorium Biologi Kesehatan | Hana Apsari Pawestri, Arie Ardiansyah Nugraha, Fajar Nur Sulistyahadi, Markus Evan  Anggia, Subangkit |  |
| EPI_ISL_19159110 | 2024-11-29 | Balai Besar Laboratorium Biologi Kesehatan | Hana Apsari Pawestri, Arie Ardiansyah Nugraha, Fajar Nur Sulistyahadi, Markus Evan  Anggia, Subangkit | Complete Genome Sequence Analysis of the First Imported Mpox Virus Clade Ib Variant in China (doi: 10.3390/pathogens14010102); Structural insights into tecovirimat antiviral activity and poxvirus resistance (https://doi.org/10.1038/s41564-025-01936-6); |
| EPI_ISL_19159111 | 2024-11-29 | Balai Besar Laboratorium Biologi Kesehatan | Hana Apsari Pawestri, Arie Ardiansyah Nugraha, Fajar Nur Sulistyahadi, Markus Evan  Anggia, Subangkit |  |
| EPI_ISL_19159112 | 2024-11-29 | Balai Besar Laboratorium Biologi Kesehatan | Hana Apsari Pawestri, Arie Ardiansyah Nugraha, Fajar Nur Sulistyahadi, Markus Evan  Anggia, Subangkit | Structural insights into tecovirimat antiviral activity and poxvirus resistance (https://doi.org/10.1038/s41564-025-01936-6); |
| EPI_ISL_19159113 | 2024-11-29 | Balai Besar Laboratorium Biologi Kesehatan | Hana Apsari Pawestri, Arie Ardiansyah Nugraha, Fajar Nur Sulistyahadi, Markus Evan  Anggia, Subangkit | Structural insights into tecovirimat antiviral activity and poxvirus resistance (https://doi.org/10.1038/s41564-025-01936-6); |
| EPI_ISL_19159114 | 2024-11-29 | Balai Besar Laboratorium Biologi Kesehatan | Hana Apsari Pawestri, Arie Ardiansyah Nugraha, Fajar Nur Sulistyahadi, Markus Evan  Anggia, Subangkit |  |
| EPI_ISL_19159115 | 2024-11-29 | Balai Besar Laboratorium Biologi Kesehatan | Hana Apsari Pawestri, Arie Ardiansyah Nugraha, Fajar Nur Sulistyahadi, Markus Evan  Anggia, Subangkit | Complete Genome Sequence Analysis of the First Imported Mpox Virus Clade Ib Variant in China (doi: 10.3390/pathogens14010102) |
| EPI_ISL_19159116 | 2024-11-29 | Balai Besar Laboratorium Biologi Kesehatan | Hana Apsari Pawestri, Arie Ardiansyah Nugraha, Fajar Nur Sulistyahadi, Markus Evan  Anggia, Subangkit |  |
| EPI_ISL_19159117 | 2024-11-29 | Balai Besar Laboratorium Biologi Kesehatan | Hana Apsari Pawestri, Arie Ardiansyah Nugraha, Fajar Nur Sulistyahadi, Markus Evan  Anggia, Subangkit |  |
| EPI_ISL_19159118 | 2024-11-29 | Balai Besar Laboratorium Biologi Kesehatan | Hana Apsari Pawestri, Arie Ardiansyah Nugraha, Fajar Nur Sulistyahadi, Markus Evan  Anggia, Subangkit |  |
| EPI_ISL_19159119 | 2024-11-29 | Balai Besar Laboratorium Biologi Kesehatan | Hana Apsari Pawestri, Arie Ardiansyah Nugraha, Fajar Nur Sulistyahadi, Markus Evan  Anggia, Subangkit |  |
| EPI_ISL_19159120 | 2024-11-29 | Balai Besar Laboratorium Biologi Kesehatan | Hana Apsari Pawestri, Arie Ardiansyah Nugraha, Fajar Nur Sulistyahadi, Markus Evan  Anggia, Subangkit | Evolutionary variation of the monkeypox virus detected for the first time in Nantong,; Jiangsu (2024-12-23); Complete Genome Sequence Analysis of the First Imported Mpox Virus Clade Ib Variant in China (doi: 10.3390/pathogens14010102); Structural insights into tecovirimat antiviral activity and poxvirus resistance (https://doi.org/10.1038/s41564-025-01936-6) |
| EPI_ISL_19336739 | 2024-11-29 | Mahidol University | Namporn,T., Manopwisedjaroen,S., Ngodngamthaweesuk,M., Pasomsub,E., Jiravejchakul,N., Saengfak,R., Nealiga,M.J.L., Sea-be,A., Basu,A., Naruphontjirakul,P.,  Hongeng,S., Tetley,T.D., Thitithanyanont,A. and Ruenraroengsak,P. |  |
| EPI_ISL_19420626 | 2024-11-29 | Research Institute for Tropical Medicine | Saul M. Rojas, Ezekiel A. Melo, Jayrecel M. Guitones, Amalea Dulcene Nicolasora, Miguel Francisco B. Abulencia, Francisco Gerardo M. Polotan, Lei Lanna M. Dancel, Timothy John  R. Dizon |  |
| EPI_ISL_19420633 | 2024-11-29 | Research Institute for Tropical Medicine | Saul M. Rojas, Ezekiel A. Melo, Jayrecel M. Guitones, Amalea Dulcene Nicolasora, Miguel Francisco B. Abulencia, Francisco Gerardo M. Polotan, Lei Lanna M. Dancel, Timothy John  R. Dizon |  |
| EPI_ISL_19437772 | 2024-11-29 | Thai Red Cross Emerging Infectious Diseases Clinical Center, King Chulalongkorn | Putcharoen,O., Rattanatumhi,K., Paitoonpong,L., Supataragul,A., Suwanpimolkul,G., Khunnawutmanotham,W., Jantarabenjakul,W., Thippamom,N., Torvorapanit,P.,  Chantasrisawad,N., Chumpa,N., Yingyong,T. and Wacharapluesadee,S. | Complete Genome Sequence Analysis of the First Imported Mpox Virus Clade Ib Variant in China (doi: 10.3390/pathogens14010102); |
| EPI_ISL_19437773 | 2024-11-29 | Thai Red Cross Emerging Infectious Diseases Clinical Center, King Chulalongkorn | Putcharoen,O., Rattanatumhi,K., Paitoonpong,L., Supataragul,A., Suwanpimolkul,G., Khunnawutmanotham,W., Jantarabenjakul,W., Thippamom,N., Torvorapanit,P.,  Chantasrisawad,N., Chumpa,N., Yingyong,T. and Wacharapluesadee,S. |  |
| EPI_ISL_19437774 | 2024-11-29 | Thai Red Cross Emerging Infectious Diseases Clinical Center, King Chulalongkorn | Putcharoen,O., Rattanatumhi,K., Paitoonpong,L., Supataragul,A., Suwanpimolkul,G., Khunnawutmanotham,W., Jantarabenjakul,W., Thippamom,N., Torvorapanit,P.,  Chantasrisawad,N., Chumpa,N., Yingyong,T. and Wacharapluesadee,S. |  |
| EPI_ISL_19437776 | 2024-11-29 | Thai Red Cross Emerging Infectious Diseases Clinical Center, King Chulalongkorn | Putcharoen,O., Rattanatumhi,K., Paitoonpong,L., Supataragul,A., Suwanpimolkul,G., Khunnawutmanotham,W., Jantarabenjakul,W., Thippamom,N., Torvorapanit,P.,  Chantasrisawad,N., Chumpa,N., Yingyong,T. and Wacharapluesadee,S. |  |
| EPI_ISL_19437777 | 2024-11-29 | Thai Red Cross Emerging Infectious Diseases Clinical Center, King Chulalongkorn | Putcharoen,O., Rattanatumhi,K., Paitoonpong,L., Supataragul,A., Suwanpimolkul,G., Khunnawutmanotham,W., Jantarabenjakul,W., Thippamom,N., Torvorapanit,P.,  Chantasrisawad,N., Chumpa,N., Yingyong,T. and Wacharapluesadee,S. |  |
| EPI_ISL_19437778 | 2024-11-29 | Thai Red Cross Emerging Infectious Diseases Clinical Center, King Chulalongkorn | Putcharoen,O., Rattanatumhi,K., Paitoonpong,L., Supataragul,A., Suwanpimolkul,G., Khunnawutmanotham,W., Jantarabenjakul,W., Thippamom,N., Torvorapanit,P.,  Chantasrisawad,N., Chumpa,N., Yingyong,T. and Wacharapluesadee,S. |  |
| EPI_ISL_19437779 | 2024-11-29 | Thai Red Cross Emerging Infectious Diseases Clinical Center, King Chulalongkorn | Putcharoen,O., Rattanatumhi,K., Paitoonpong,L., Supataragul,A., Suwanpimolkul,G., |  |
| EPI_ISL_19437780 | 2024-11-29 | Thai Red Cross Emerging Infectious Diseases Clinical Center, King Chulalongkorn | Putcharoen,O., Rattanatumhi,K., Paitoonpong,L., Supataragul,A., Suwanpimolkul,G., Khunnawutmanotham,W., Jantarabenjakul,W., Thippamom,N., Torvorapanit,P.,  Chantasrisawad,N., Chumpa,N., Yingyong,T. and Wacharapluesadee,S. |  |
| EPI_ISL_19456511 | 2024-11-29 | Research Institute for Tropical Medicine | Saul M. Rojas, Ezekiel A. Melo, Jayrecel M. Guitones, Samantha Louise P. Bado, Chelsea Mae M. Reyes, Amalea Dulcene Nicolasora, Miguel Francisco B. Abulencia, Francisco  Gerardo M. Polotan, Lei Lanna M. Dancel, Timothy John R. Dizon |  |
| EPI_ISL_19456608 | 2024-11-29 | Research Institute for Tropical Medicine | Saul M. Rojas, Ezekiel A. Melo, Jayrecel M. Guitones, Samantha Louise P. Bado, Chelsea Mae M. Reyes, Amalea Dulcene Nicolasora, Miguel Francisco B. Abulencia, Francisco  Gerardo M. Polotan, Lei Lanna M. Dancel, Timothy John R. Dizon |  |
| EPI_ISL_13056274 | 2024-11-29 | Centers for Disease Control and Prevention | Mauldin,M.R., McCollum,A.M., Nakazawa,Y.J., Mandra,A., Whitehouse,E.R., Davidson,W.,  Zhao,H., Gao,J., Li,Y., Doty,J., Yinka-Ogunleye,A., Akinpelu,A., Aruna,O., Naidoo,D.,  Lewandowski,K., Afrough,B., Graham,V., Aarons,E., Hewson,R., Vipond,R., Dunning,J.,  Chand,M., Brown,C., Cohen-Gihon,I., Erez,N., Shifman,O., Israeli,O., Sharon,M.,  Schwartz,E., Beth-Din,A., Zvi,A., Mak,T.M., Ng,Y.K., Cui,L., Lin,R.T.P., Olson,V.A.,  Brooks,T., Paran,N., Ihekweazu,C. and Reynolds,M.G. | Global genomic surveillance of monkeypox virus (doi: 10.1038/s41591-024-03370-3 ); Phylogenomic and Structural Analysis of the Monkeypox Virus Shows Evolution towards; Increased Stability (doi: 10.3390/v15010127 ); Molecular Evolution of Protein Sequences and Codon Usage in Monkeypox Viruses (https://doi.org/10.1093/gpbjnl/qzad003); Comparative genome analysis reveals driving forces behind Monkeypox virus evolution and sheds light on the role of ATC trinucleotide motif (https://doi.org/10.1093/ve/veae043); A comprehensive review of monkeypox virus and mpox characteristics (https://doi.org/10.3389/fcimb.2024.1360586) |
| EPI_ISL_19437775 | 2024-11-29 | Thai Red Cross Emerging Infectious Diseases Clinical Center, King Chulalongkorn | Putcharoen,O., Rattanatumhi,K., Paitoonpong,L., Supataragul,A., Suwanpimolkul,G.,  Khunnawutmanotham,W., Jantarabenjakul,W., Thippamom,N., Torvorapanit,P.,  Chantasrisawad,N., Chumpa,N., Yingyong,T. and Wacharapluesadee,S. |  |
